# VG-RETFound: a hybrid retinal AI framework for early vision impairment screening through vessel-graph learning and cross-modal fusion

**DOI:** 10.3389/fmed.2026.1905129

**Published:** 2026-09-01

**Authors:** Ashit Kumar Dutta, Nasser Ali Aljarallah, Abdul Rahaman Wahab Sait

**Affiliations:** 1 Department of Computer Science and Information Systems, College of Applied Sciences, AlMaarefa University, Riyadh, Saudi Arabia; 2 King Salman Center for Disability Research, Riyadh, Saudi Arabia; 3 Department of Documents and Archive, Center of Documents and Administrative Communication, King Faisal University, Al Hofuf, Al-Ahsa, Saudi Arabia

**Keywords:** cross-modal attention fusion, explainable artificial intelligence, graph attention network, RETFound foundation model, retinal disease classification, vision impairment screening

## Abstract

**Introduction:**

Vision impairment caused by retinal diseases stands out as one of the significant sources of irreversible blindness, highlighting the importance of developing robust automated screening tools to detect eye diseases at an early stage. Despite the promising results of recent deep learning approaches to the automatic classification of retinal images, most existing solutions focus on image-based representations and do not consider the importance of vascular topology. To overcome the limitations, this study proposes VG-RETFound – a hybrid framework based on a retinal foundation model and graph-based vascular-topology modeling that can be used to classify multiple retinal categories and detect vision impairment.

**Methods:**

The authors use RETFound—a retinal foundation model—pretrained on a vast number of retinal images, as well as the Graph Attention Network (GAT), to learn vascular topology features by representing retinal vessels as graphs. The presented approach employs a cross-modal multi-head attention fusion method to integrate features from both modalities in real time. To evaluate the efficiency of VG-RETFound, experiments were conducted using the RFMiD and ODIR-5 K datasets for model training and testing across six retinal categories: Normal, Diabetic Retinopathy, Pathological Myopia, Age-related Macular Degeneration, Hypertensive Retinopathy, and Other Retinal Diseases.

**Results:**

The accuracy of 96.39%, F1-score of 95.84%, MCC of 94.87%, and Kappa of 94.71% were attained on an internal test dataset, while cross-dataset external validation on the BRSET dataset revealed accuracy of 94.81%, F1-score of 94.25%, MCC of 93.09%, and Kappa of 92.94%.

**Discussion:**

These findings demonstrate that integrating retinal foundation models with anatomically informed vessel-topology learning significantly improves classification accuracy, interpretability, and generalization, highlighting the potential of VG-RETFound as a tool for large-scale vision impairment. screening and retinal disease diagnosis.

## 1 Introduction

Vision impairment remains a major global health concern, affecting millions of individuals worldwide and significantly reducing quality of life, productivity, and independence (1–3). Among the leading causes of permanent visual impairment, international public health and ophthalmologic organizations highlight the presence of diabetic retinopathy (DR), glaucoma, age-related macular degeneration (AMD), hypertensive retinopathy (HTR), and pathological myopia (PM) (4–6). The prevalence rates of these disorders increase sharply due to aging populations, higher rates of diabetes, and various diseases resulting from individuals’ lifestyles (7). An important characteristic of diseases affecting the retina is their gradual development and absence of symptoms in the early stages, causing patients to seek medical attention only after significant retinal damage has occurred (8). Consequently, early detection and timely intervention are critical for preventing vision impairment.

Fundus image is considered one of the most widely used modalities for diagnosing and screening for ophthalmic conditions due to its non-invasiveness and cost-effectiveness compared with other imaging techniques (9, 10), providing valuable clinically relevant information about the optic disc, macula, retinal vasculature, hemorrhages, exudates, drusen, and other ocular structures related to various diseases and systemic conditions. Ophthalmologists manually analyze fundus images and perform clinical assessments of the diseases. Although this approach is commonly used in ophthalmic practice, it has significant limitations, as it requires substantial time and resources (11–13). Moreover, the subjective and observer-dependent interpretation of fundus photographs may be influenced by inter-rater variability, particularly in the case of mild disease progression (14). These limitations have driven the need for automated computer-assisted diagnosis of retinal disease using artificial intelligence (AI) techniques.

In recent years, AI techniques have attracted increasing interest in medical image analysis and hold great promise in the diagnosis of retinal diseases (15–17). Deep learning-driven diagnostic systems automatically extract disease-related features and classify or grade diseases from image data (18). Traditional AI-based systems for retinal diagnostics used handcrafted features, including texture descriptors, edge information, color histograms, vessel morphology, and morphological operations (19). Classification of retinal images using handcrafted features was performed with traditional machine learning algorithms, including support vector machines, decision trees, random forests, and k-nearest neighbors (20). Although such techniques showed promising results, they were limited in terms of their ability to generalize and lacked clinical relevance.

The development of deep learning architectures, specifically convolutional neural networks (CNNs), has led to significant improvements in retinal image analysis (21). CNNs architectures have demonstrated strong performance on various retinal analysis tasks, including DR grading, glaucoma detection, optic disc segmentation, lesion localization, and multiple retinal disease classification (22). Compared with traditional machine learning algorithms, deep learning CNNs can learn complex feature hierarchies from retinal images (23). Transfer learning strategies using models pre-trained on large-scale natural image datasets further improved performance, particularly when labeled medical datasets were limited. Vision transformers (ViTs) have become a powerful alternative to traditional CNN architectures (21, 24). By introducing self-attention mechanisms, transformers enable learning of global relationships among elements in the data sample. ViTs have shown strong results in several medical tasks, including retinal disease classification and segmentation, as well as lesion localization. Recently, retinal foundation models have been introduced to pretrain a network on a large-scale dataset of retinal images using self-supervised learning to learn generalized retinal representations (25).

Despite significant advances in developing AI-based diagnostic solutions for retinal disorders, several critical limitations remain. For instance, most deep learning models used in this field rely on image pixel representations and fail to incorporate clinically important anatomical information during training. Retinal vascular structures are highly important biomarkers for numerous retinal and systemic diseases. Variations in vessel tortuosity, branching patterns, vessel caliber, and neovascularization are strongly associated with the progression of DR, HTR, retinal vascular occlusions, and glaucoma-related changes. However, standard CNN and transformer architectures are generally expected to learn such vascular characteristics implicitly through feature optimization. This indirect learning process may reduce sensitivity to subtle structural abnormalities and limit interpretability from a clinical perspective.

Another drawback of current approaches to retinal disease classification is their poor performance on multi-disease or multi-class classification tasks (26). Individuals may have multiple retinal pathologies simultaneously, complicating disease classification and posing challenges for their coexistence. Existing retinal AI diagnostic models are usually designed for single-disease classification and do not account for possible disease co-occurrence. Another problem is that retinal image datasets are often highly imbalanced because some diseases occur less frequently than others, making it challenging to detect less frequent retinal pathologies.

Generalization of models remains a key issue in AI research, as even state-of-the-art CNNs and transformers exhibit reduced performance on unseen datasets due to variations in illumination, field of view, camera type, color balance, and other factors. Despite the use of data augmentation, transfer learning, and self-supervised learning, deep learning-based retinal AI models still fail to achieve satisfactory results across diverse datasets. Among retinal imaging modalities, fundus photography-based AI systems offer several practical advantages over alternative methods, including optical coherence tomography (OCT), fluorescein angiography, and invasive diagnostic procedures. Fundus photography, by contrast, is inexpensive, non-invasive, and requires no specialized equipment or trained personnel, making fundus-based AI models suitable for widespread implementation and population screening.

Although fundus-image-based deep learning systems have demonstrated strong performance, most existing models still rely exclusively on appearance-based representations and do not explicitly model retinal anatomical structures. Recent studies have explored graph neural networks, vessel segmentation approaches, and multimodal fusion techniques to incorporate structural information into retinal analysis. However, most of these approaches are applicable only to vessel segmentation or retinal disease classification. Additionally, most approaches to multimodal fusion employ late fusion or concatenation of features and cannot represent relationships between global image context and local structural vessel topology. These limitations motivated the development of an integrated framework capable of simultaneously exploiting global retinal image representations, structural vascular information, efficient model adaptation, and interpretable decision-making. This study aims to develop a robust, anatomically informed, and computationally efficient AI framework for automated classification of multi-disease retinal images.

The study contributions are as follows:

- A novel hybrid vision impairment screening architecture combining RETFound-based global feature learning with graph-based vessel topology modeling, enabling the model to jointly learn appearance-level and anatomical representations from the fundus images.
- A cross-modal multi-head attention fusion mechanism that fuses RETFound image tokens and vessel-graph embeddings, allowing the model to identify complex structures that are most relevant to disease-specific retinal patterns.
- An asymmetric structural fine-tuning paradigm is developed, in which the large RETFound backbone is adapted using parameter-efficient fine-tuning, while the vessel-graph module, cross-attention fusion layers, and classification head are comprehensively fine-tuned, improving computational efficiency while preserving critical retinal features associated with normal and abnormal classes.

The innovation of the proposed framework lies in integrating the representation-learning capability of a retinal foundation model with explicit modeling of anatomical vessel topology. Specifically, retinal vessels are extracted using a pre-trained vessel segmentation model, and the segmented vasculature is converted into a graph representation that encodes structural information, including vessel connectivity, bifurcation patterns, orientation, and tortuosity. These graph representations are processed using a Graph Attention Network (GAT), enabling the model to learn clinically meaningful vascular relationships.

The scope of this study is limited to automated multi-class retinal disease classification using color fundus images. The proposed framework is designed to classify six retinal categories, including normal retina, diabetic retinopathy, pathological myopia, age-related macular degeneration, hypertensive retinopathy, and other retinal diseases. The study focuses on improving classification performance, robustness, cross-dataset generalization, and interpretability through integrated multimodal representation learning.

The rest of this study is structured as follows. Section 2 describes the materials and methods utilized in this study, which include dataset collection, data pre-processing, the proposed VG-RETFound model, vessel graph generation, the multimodal feature fusion approach, and the asymmetric structure fine-tuning process. In Section 3, the experimental results of the proposed model and their evaluation are described, with an emphasis on classification accuracy and comparisons with other models. The discussion of the importance of the obtained results, the benefits of the proposed method, clinical significance, limitations, and perspectives for future work is provided in Section 4. Finally, Section 5 concludes the study by summarizing the major contributions and highlighting the proposed framework's potential for automated diagnosis of vision impairment.

## 2 Literature review

Recent AI systems have demonstrated considerable success in classifying retinal diseases, including DR, age-related macular degeneration (AMD), PM, HTR, and other retinal disorders (25). Among these approaches, CNNs and ViTs have become the dominant paradigms for retinal image classification.

CNN-based models, including ResNet, DenseNet, EfficientNet, and Inception architectures, learn hierarchical feature representations through stacked convolutional operations (21, 26, 27). Early layers extract low-level image features such as edges, vessel boundaries, and texture, whereas deeper layers capture disease-specific characteristics, including hemorrhages, exudates, drusen, and optic disc abnormalities. Owing to their strong local feature extraction capability, CNNs have achieved remarkable performance in retinal disease classification and have been extensively adopted in automated screening systems (21). Furthermore, transfer learning from large-scale natural image datasets has improved classification accuracy when labeled retinal datasets are limited.

CNNs typically learn vascular information implicitly from image pixels rather than explicitly modeling retinal vessel structures (21). Consequently, subtle vascular abnormalities such as vessel tortuosity, narrowing, and branching irregularities may not be adequately represented. These limitations can reduce the effectiveness of CNNs-based systems in identifying diseases where vascular pathology plays a critical diagnostic role. To overcome the restricted contextual modeling capability of CNNs, ViTs have been increasingly applied to retinal image analysis (28). Unlike CNNs, which rely on local convolutional filters, self-attention enables ViTs to capture global contextual information and long-range dependencies across the retina (29). This capability is particularly beneficial for retinal disease classification because pathological manifestations may occur across multiple retinal regions rather than being confined to a single localized area. Recent transformer-based models have demonstrated improved performance in multi-class retinal disease classification and have shown superior representation learning compared with conventional CNN architectures.

Although ViTs address several limitations of CNNs, they present notable challenges. ViTs remain fundamentally appearance-based models that operate on image patches. While they can identify disease-relevant regions, they do not explicitly encode anatomical relationships or structural connectivity within the retinal vasculature (30). As a result, clinically important vascular biomarkers may not be optimally represented. Furthermore, transformer-based architectures are computationally demanding, particularly when fine-tuning large pretrained models for downstream ophthalmic applications.

To improve retinal disease classification, recent studies have explored hybrid architectures that combine CNNs and transformers (31). These approaches aim to exploit the complementary strengths of local feature extraction and global contextual learning. Although hybrid models have achieved promising results, most employ simple feature concatenation or late-fusion strategies to combine different feature representations (32). Such fusion mechanisms often assume independent contributions from each modality and fail to model the complex interactions between retinal lesions and vascular abnormalities. Consequently, important anatomical relationships may remain underutilized during classification.

The emergence of retinal foundation models, particularly RETFound, represents a significant advancement in ophthalmic AI (33–35). However, despite its strong global feature learning capability, RETFound primarily focuses on appearance-based retinal representations and does not explicitly model retinal vascular topology. Since vascular abnormalities constitute key indicators of many vision-threatening diseases, relying solely on image-level representations may limit diagnostic performance.

The retinal vessels form an interconnected system that can be modeled as a graph structure. Graph neural networks, especially GAT, provide an explicit approach to modeling vessel connectivity, branching, and vascular topography (36). Table 1 compares recent deep learning approaches for ocular disease classification, highlighting their methodologies, major contributions, and existing limitations. The reviewed studies demonstrate significant advances through convolutional neural networks, Vision Transformers, feature-fusion techniques, explainable AI, federated learning, and graph neural networks.

**Table 1:** Summary of recent deep learning approaches for ocular disease classification.

Study	Method	Main contribution	Key limitation
Al-Fahdawi et al. (28)	Fundus-DeepNet	Multi-class classification using fundus-image data fusion	No explicit vessel-topology modeling
Ouda et al. (29)	Multi-class CNN	Simultaneous diagnosis of multiple ocular diseases	Lacks foundation-model pretraining and graph learning
Sengar et al. (30)	Eye Deep-Net	Multi-class retinal disease classification	Primarily image-based; no structural vessel representation
Venkataiah et al. (31)	Segmentation–classification model	Combines lesion segmentation with disease classification	Limited evidence of vessel-graph or cross-modal fusion
Ali et al. (32)	Tuned Vision Transformer with XAI	Global feature learning with explainable predictions	Thesis-based and image-centric
Nazih et al. (33)	Vision Transformer	Diabetic-retinopathy severity prediction	Limited to a single disease
Jayalakshmi et al. (34)	Federated-learning models	Privacy-preserving distributed disease detection	Not specifically designed for retinal anatomy
Purba et al. (35)	Vision Transformer	Transformer-based eye-disease classification	No vessel graph or foundation-model adaptation
Sathiya et al. (36)	CNN–Transformer framework	Combines local and global retinal features	No explicit vascular-topology modeling
Sah et al. (37)	EfficientNetB0 fusion	Multi-model feature fusion for eye-disease classification	Uses homogeneous CNN features rather than image–graph fusion
Nirob et al. (38)	EDDNet30	Spatial attention, multiscale fusion, and XAI	Does not explicitly model vessel connectivity
Alqassab et al. (39)	Hybrid quantum CNN	Quantum–classical ocular-disease classification	Higher implementation complexity and no vessel-graph learning
Inbakumar and Illakiyavarshini (40)	GCN, GAT, and GraphSAGE	Graph-based eye-disease detection	Does not integrate graph features with a retinal foundation model

Although the existing methods achieve promising classification performance, they primarily rely on image-based representations and do not explicitly capture retinal vascular topology or integrate graph-based structural information with foundation models. In addition, several approaches are limited to specific diseases or lack adaptive multimodal feature fusion. These limitations led to the development of the proposed framework, integrating retinal foundation-model representations, vessel graph learning, and cross-modal attention for comprehensive retinal disease screening.

## 3 Materials and methods

**Figure 1:**
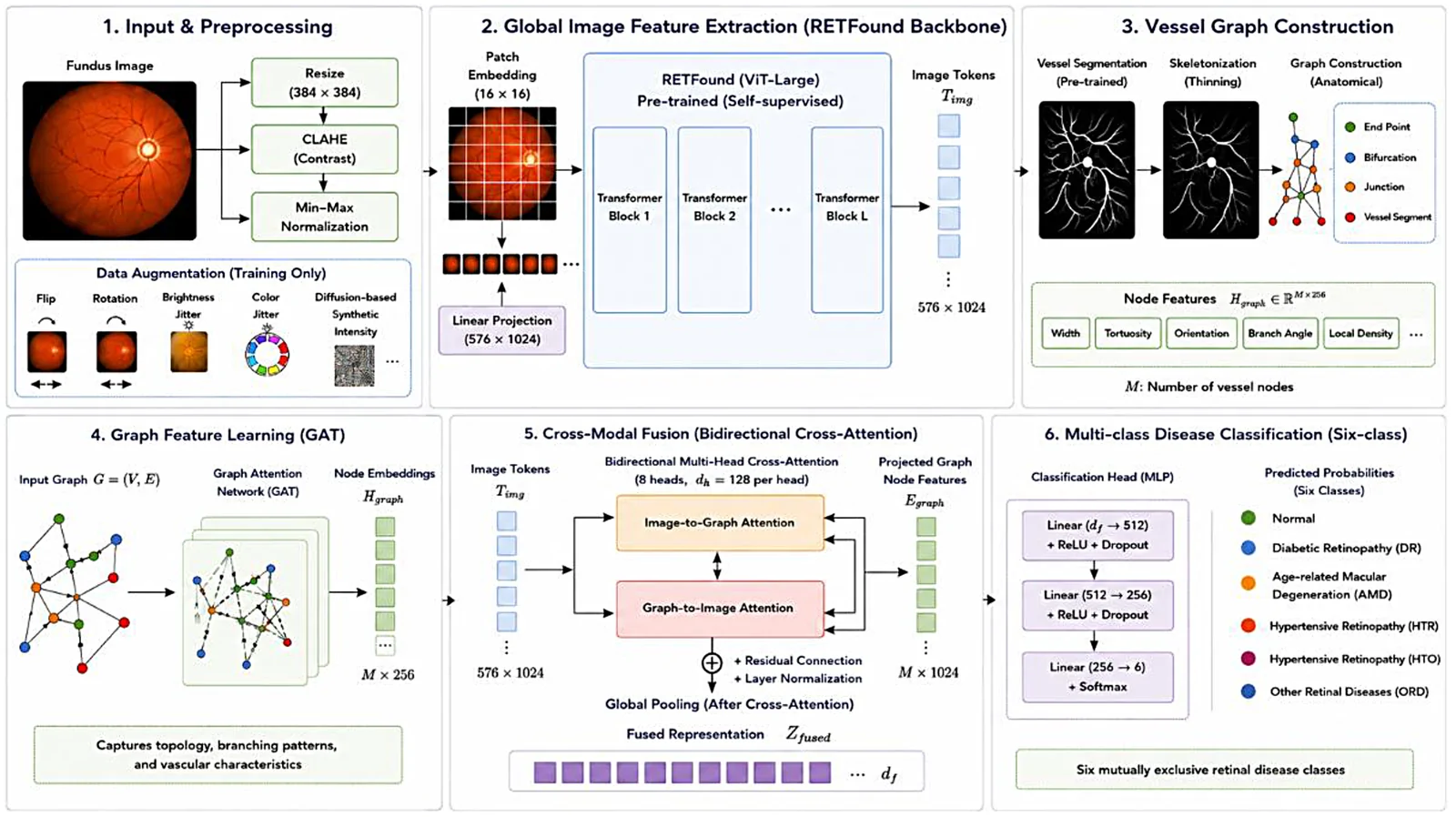
The proposed early vision impairment screening approach.

Figure 1 shows the architecture of the proposed VG-RETFound framework. The overall design aims to overcome the drawbacks of traditional retinal disease classification models by explicitly incorporating global retinal representation and vessel topology information into the model. The proposed method starts with the preprocessing and augmentation of the fundus images. Following the data preprocessing step, RETFound backbone extracts global features from the retinal images. This backbone was selected for its ability to learn more robust representations through self-supervised learning.

To leverage clinically relevant information, the next stage of the proposed framework includes the retinal vessels segmentation with the subsequent transformation into the graph representation to learn vessel connectivity, branching patterns, tortuosity, and orientation characteristics. GAT learns structural vascular relationships, as graph-based learning effectively models interconnected anatomical structures that are difficult to represent using traditional image-based approaches. Learned graph embeddings and image embeddings extracted by RETFound model are merged together via multi-head cross-modal attention fusion to allow interactions between global retinal context and local vessel topology representations.

Algorithm 1 presents the overall implementation pipeline of the proposed VG-RETFound framework, outlining the sequential stages from dataset preparation to final performance evaluation. The workflow emphasizes a structured learning process in which model development, optimization, and validation are conducted under a unified experimental protocol. By clearly separating model training from independent evaluation, the algorithm illustrates how the proposed framework ensures reliable performance estimation while preserving the integrity of the validation process. This systematic pipeline also facilitates reproducibility by providing a transparent overview of the key computational stages involved in feature learning, multimodal fusion, and generalization assessment across internal and external datasets.

Algorithm 1 **Data Preparation, Leakage Prevention, and External Validation**

**Input:**

** RFMiD and ODIR development datasets**

**BRSET external validation dataset**

**Output:**

** Trained VG-RETFound model and evaluation results**

**1: Combine RFMiD and ODIR development data.**

**2: Group all images by patient identifier.**

**3: Split patients into five non-overlapping folds.**

**4: for each cross-validation fold do**

**5: Assign patient groups to training, validation, and test subsets.**

**6: Ensure that no patient appears in more than one subset.**

**7: Apply preprocessing independently to each subset.**

**8: Apply random rotation, flipping, brightness adjustment,**

** and color jitter only to the training subset.**

**9: Generate diffusion-based synthetic samples only for**

** minority classes in the training subset.**

**10: Train VG-RETFound using the augmented training data.**

**11: Select model settings using the validation subset.**

**12: Evaluate the final fold model on the unchanged test subset.**

**13: end for**

**14: Aggregate the performance metrics across the five folds.**

**15: Apply the finalized preprocessing procedure to BRSET.**

**16: Evaluate the trained model on BRSET without augmentation,**

** retraining, or parameter tuning.**

**17: Report internal and external validation results.**

### 3.1 Dataset description

In this study, three public fundus retinal image datasets, including RFMiD (41), ODIR-5 K (42), and BRSET (43), are used to ensure comprehensive evaluation of performance over various disease types, imaging modalities, and population groups. Specifically, the RFMiD database consists of 3,200 color fundus images that are associated with 46 types of retinal diseases and constitutes one of the largest collections for multi-disease retinal classification. Being multilabel and highly imbalanced, this database provides an appropriate testing ground for the framework to demonstrate its ability to detect both frequently encountered and rare retinal abnormalities. Moreover, the ODIR-5 K database comprises approximately 10,000 retinal images from 5,000 individuals and includes information on eight major retinal abnormalities, namely diabetic retinopathy, glaucoma, cataract, age-related macular degeneration, and pathological myopia. The significant variability in image quality in this dataset allows evaluation of the framework's efficiency in real-world clinical settings. Finally, the BRSET dataset contains 16,266 high-quality retinal fundus images annotated with multi-class retinal diseases from the Brazilian population. Collectively, these datasets provide a broad spectrum of vision-impairing diseases, thereby establishing a rigorous benchmark for evaluating the proposed VG-RETFound framework in automated multi-disease retinal diagnosis. Table 2 presents the key characteristics of the datasets utilized in this study and their respective contributions to the evaluation of the proposed VG-RETFound framework.

**Table 2:** Characteristics of the datasets used for evaluating the proposed VG-RETFound framework.

Dataset	Geographic Origin	Annotation Type	Clinical Complexity	Imaging Diversity	Multi-class Support	Role in VG-RETFound Evaluation
RFMiD (41)	India	Disease-specific retinal annotations	High (multiple co-existing retinal abnormalities)	Moderate	Yes	Evaluation of multi-disease recognition and class imbalance handling
ODIR-5 K (42)	China	Patient-level ocular disease labels	Moderate to High	High (multiple cameras and acquisition settings)	Yes	Assessment of robustness and domain adaptation capability
BRSET (43)	Brazil	Multi-class retinal disease annotations with associated clinical information	High	High	Yes	External Validation

### 3.2 Dataset harmonization and class selection strategy

Since RFMiD, ODIR-5 K, and BRSET employ different disease taxonomies and annotation schemes, their original annotations were harmonized into a unified six-class mutually exclusive multiclass classification problem comprising Normal, Diabetic Retinopathy (DR), Age-related Macular Degeneration (AMD), Pathological Myopia (PM), Hypertensive Retinopathy (HTR), and Other Retinal Diseases (ORD). Accordingly, each fundus image was assigned exactly one target class, and the classification head produced a single probability distribution across the six classes using Softmax activation.

Equivalent diagnostic annotations were first mapped to their corresponding harmonized categories. Diabetic-retinopathy-related labels were mapped to DR, age-related macular degeneration labels to AMD, pathological-myopia or myopic-fundus labels to PM, and hypertensive-retinopathy labels to HTR. Retinal abnormalities that could not be consistently represented as one of these four target diseases—including glaucoma, retinal vein occlusion, optic-disc abnormalities, drusen, macular hole, cataract, and other less frequently represented disorders—were grouped into ORD. An image was assigned to Normal only when no pathological annotation was present.

For images containing multiple original disease labels, a deterministic hierarchical assignment rule was applied to obtain a single harmonized label. The priority order was defined as DR > AMD > PM > HTR > ORD > Normal. Thus, when multiple target conditions co-occurred, the image was assigned to the highest-priority disease present according to this hierarchy. For example, an image annotated with both DR and AMD was assigned to DR, whereas an image containing AMD together with an abnormality belonging to ORD was assigned to AMD. Images containing only non-target retinal abnormalities were assigned to ORD, while Normal was used exclusively for images without any disease annotation. The same mapping and priority rules were applied consistently to RFMiD, ODIR-5 K, and the BRSET external-validation subset, ensuring that every image contributed to only one class and preventing duplicate assignment across categories. For BRSET, harmonization was performed only at the annotation level to make its original multilabel annotations compatible with the six-class evaluation protocol. No training augmentation, synthetic image generation, hyperparameter tuning, or model optimization was performed using the external-validation data. The resulting harmonized class distributions for model development and external validation are summarized in Table 3.

**Table 3:** Label harmonization strategy and final image distribution across the development and external validation datasets.

Harmonized Class	RFMiD Labels	ODIR-5 K Labels	BRSET Labels	Combined RFMiD + ODIR Images	BRSET Images (20%)
Normal	Normal	Normal	Normal	4,151	1,692
DR	DR	Diabetes (D)	diabetic_retinopathy	1,984	209
AMD	AMD	AMD (A)	amd	387	73
PM	Pathological Myopia	Myopia (M)	myopic_fundus	348	54
HTR	Hypertensive Retinopathy	Hypertension (H)	hypertensive_retinopathy	168	57
ORD	Remaining retinal diseases	Other abnormalities (O)	other	1,977	151
Total Images				9,015	2,236

Following label harmonization, the combined RFMiD–ODIR dataset was used for model development through five-fold stratified cross-validation. The harmonized BRSET pool contained 11,181 eligible fundus images. For independent external validation, a stratified 20% subset comprising 2,236 images was selected from this pool while approximately preserving the harmonized class distribution. The resulting external cohort contained 1,692 Normal, 209 DR, 73 AMD, 54 PM, 57 HTR, and 151 ORD images. BRSET was not used for model training, augmentation, synthetic data generation, hyperparameter tuning, or model selection, thereby maintaining separation between model development and external performance assessment.

The BRSET dataset was used solely as an independent external-validation. The reported mean ± standard deviation (SD) was obtained by evaluating the same fixed 20% BRSET subset with independently trained instances of the proposed model generated during the internal training procedure. For each model instance, Accuracy, Precision, Recall, F1-score, MCC, and Kappa were calculated on the identical external cohort. The mean for each metric represents the average performance across these repeated independent evaluations, while the SD quantifies the variation in external performance caused by differences in model initialization and training. Therefore, the reported mean ± SD reflects model-level variability on a fixed external dataset, rather than variability across BRSET folds.

### 3.3 Input image preprocessing and augmentation

Image preprocessing and augmentation were introduced to minimize intra-dataset variance and improve the stability of feature learning. Since the RFMiD and ODIR-5 K datasets were captured using different imaging machines, acquisition procedures, and illumination environments, the raw fundus images exhibit varying degrees of heterogeneity, which can negatively affect model performance. In order to guarantee uniformity between different datasets and be consistent with the input size of the RETFound backbone, all images were resized to 384 × 384 pixels. Subsequently, Contrast Limited Adaptive Histogram Equalization (CLAHE) was applied to boost the local contrast and enhance the visibility of important retinal features like blood vessels, microaneurysms, hemorrhages, and exudates. Normalization of the pixel values was also adopted to account for possible illumination changes and enable effective model training.

To enhance data diversity and improve model robustness, several data augmentation techniques were applied to the training set, including random rotation, horizontal and vertical flipping, brightness adjustment, and color jitter. In addition, diffusion model-based augmentation was employed to generate synthetic fundus images for underrepresented disease classes, thereby mitigating class imbalance and improving representation of minority categories. These augmentation procedures were applied only to the training data after dataset splitting, while the validation and test sets remained unaltered to ensure an unbiased evaluation. To prevent data leakage, dataset partitioning was performed before any augmentation or preprocessing that could introduce information overlap across subsets. Consequently, no original or synthetically generated images from the training set were included in the validation or test sets. Furthermore, patient-level separation was maintained throughout the data splitting process, ensuring that images from the same patient were assigned exclusively to a single subset. This strategy prevented information leakage arising from multiple images of the same individual.

All preprocessing and augmentation procedures were performed exclusively on the combined RFMiD–ODIR dataset used for model development and internal five-fold cross-validation. The BRSET dataset was reserved exclusively for external validation and was not used during model training, data augmentation, hyperparameter tuning, or model selection. The BRSET dataset was reserved exclusively for external validation and was not used during model training, data augmentation, hyperparameter tuning, or model selection. To ensure compatibility with the proposed framework, only deterministic image preprocessing operations required during inference, including image resizing, CLAHE enhancement, and intensity normalization, were applied to the BRSET images. In addition, only labeling inconsistencies were harmonized to conform to the unified six-class multi-class classification protocol, ensuring consistent class definitions across all datasets without modifying the original image content. No training-dependent preprocessing, data augmentation, synthetic image generation, or parameter optimization was performed on the external dataset, thereby preserving its independence for an unbiased assessment of the generalization capability of the proposed VG-RETFound framework.

### 3.4 Global retinal representation learning using RETFound

Global representation learning in the VG-RETFound framework is achieved using RETFound, the retinal foundation model, which was used primarily for the derivation of global representations of the retina based on the learned retinal properties and pathology patterns. RetFound was selected as the core visual backbone because it is pre-trained on around 1.6 million retinal images based on self-supervised masked autoencoding learning, allowing the model to discover retinal-related properties and patterns automatically without the requirement of manually annotated training samples. Compared with the typical deep learning models, especially CNNs, which focus more on local spatial characteristics of data samples and require large amounts of labeled data for effective learning, RETFound has demonstrated strong generalizability in learning general representations of the retina using large-scale retinal data and can perform well across different tasks within the ophthalmological field. This characteristic makes RETFound an ideal choice in the current research, where retinal images are acquired from multiple datasets with varying distributions of diseases, acquisition protocols, and imaging devices.

RETFound leverages the ViT framework by breaking down the retinal image into patches and transforming them into the latent embeddings passed further through several layers of self-attention modules. The self-attention mechanism enables the model to capture contextual relationships among anatomically distant retinal regions, allowing simultaneous characterization of optic disc morphology, macular structures, vascular patterns, hemorrhages, exudates, and other pathological manifestations. Such global contextual learning provides a significant advantage over conventional CNN-based approaches, as retinal abnormalities associated with vision impairment are often distributed across multiple retinal regions rather than being confined to localized structures. Equation 1 expresses the retinal representation extracted through the RETFound model.

$$T_{img}=Transformer(PatchEmbed(I))$$ (1)

where *I* denotes the input retinal image, PatchEmbed(.) represents the patch embedding operation, transforming image patches into token embeddings, Transformer(.) denotes the stacked self-attention encoder layers, and $T_{img}$ indicates the high-level retinal representation. This embedding serves as the global appearance-based modality in our proposed framework, providing full retinal context for the disease diagnosis. While RETFound captures retinal appearance information and certain vascular characteristics, it lacks the ability to capture any topological structure of the retinal vascular network. Therefore, an additional vessel-graph learning branch is incorporated to capture complementary vascular information and enhance the diagnosis of vision-threatening retinal diseases.

### 3.5 Vessel segmentation and graph construction

Vascular abnormalities in the retina serve as essential biomarkers for multiple eye conditions that affect vision, including diabetic retinopathy, glaucoma, hypertensive retinopathy, retinal vein occlusion, and age-related vascular diseases. Even though transformer-based methods can capture vessel attributes automatically during training, they fail to maintain relationships among vascular segments. Therefore, the vessel topology learning mechanism is added to the proposed architecture, which models vessels as graphs. Initially, a pre-trained model for the task of retinal vessel segmentation is used to obtain the vascular structures in the fundus image. The use of a pre-trained vessel segmentation model eliminates the requirement for manual labeling of retinal vessels in the target datasets. Equation 2 represents the segmentation output.

$$M_{v}=f_{seg}(I)$$ (2)

where $M_{v}$ denotes the binary vessel mask. Following segmentation, skeletonization is applied to reduce vessel structures to their centerlines while preserving vascular connectivity and branching patterns, facilitating efficient graph construction and removing redundant pixel information.

This vascular network is then modeled as a graph *G* *=* *(V, E)*, where the nodes represent vessel endpoints, branch points, and vessel segments, and the edges represent anatomical connectivity among adjacent structures. Unlike image-based modeling, the graph-based approach explicitly captures the topology of vessel components. Graph nodes are extracted from three vascular components: vessel endpoints, bifurcation points, and vessel segments. Endpoints are identified as terminal pixels with a single neighboring vessel pixel, whereas bifurcation points correspond to junctions connected to three or more neighboring vessel pixels. Vessel segments between adjacent junctions are represented as graph components that preserve the continuity of the vascular tree.

Edges are established according to the anatomical connectivity of the retinal vasculature. Two nodes are connected whenever they belong to the same continuous vessel segment without an intervening branching point or endpoint, thereby preserving the natural topology of the retinal vascular network rather than relying solely on spatial proximity.

To characterize each node, a feature vector is constructed using five vascular descriptors: vessel width, tortuosity, orientation, branch angle, and local vessel density. These anatomical features capture both local vessel morphology and structural connectivity, providing complementary information to the global retinal representations extracted by RETFound.

Z-score normalization is used to normalize the node feature as shown in Equation 3.

$$\hat{x}=\;\frac{x-\mu }{\sigma +\epsilon }$$ (3)

Where μ and σ denote the mean and standard deviation computed from the training data and ϵ is a small constant added for numerical stability. The normalized node feature matrix, together with the adjacency matrix describing vessel connectivity, forms the graph representation that serves as the input to the Graph Attention Network (GAT) described in the following subsection.

### 3.6 Vessel topology feature learning using GAT

After constructing the retinal vessel graph, the proposed framework employs a three-layer Graph Attention Network (GAT) with eight attention heads to learn discriminative vascular representations. Unlike conventional Graph Convolutional Networks (GCNs), which aggregate neighboring node information using fixed weights, GAT dynamically assigns attention coefficients to neighboring vessel structures. This adaptive mechanism enables the model to emphasize clinically significant vascular patterns that are more informative for retinal disease diagnosis.

For a target node *i*, the attention coefficient between node i and its neighboring node *j* is computed as shown in Equation 4.

$$\alpha_{ij}=\frac{\exp(\mathrm{LeakyReLU}(a^{T}[Wh_{i}\;||\;Wh_{j}]))}{\sum_{k\in N(i)}\exp(\mathrm{LeakyReLU}(a^{T}[Wh_{i}\;||\;Wh_{k}]))}$$ (4)

where $h_{i}$ and $h_{j}$ denote the feature representations of nodes i and *j*, respectively, *W* represents a learnable linear transformation matrix, *a* is the attention weight vector, N(i) denotes the neighborhood of node *i*, and ∣∣ indicates vector concatenation. The resulting attention coefficient $\alpha_{ij}$ quantifies the relative importance of neighboring vessel structures during feature aggregation. Consequently, vessel segments exhibiting clinically significant abnormalities, such as excessive tortuosity, vessel narrowing, irregular branching, or abnormal vascular dilation, can receive higher attention weights and contribute more substantially to the learned representation.

The attention-weighted node features are iteratively aggregated through multiple GAT layers, enabling local vascular characteristics to propagate throughout the retinal vascular network and capture higher-order structural relationships. This hierarchical aggregation allows the network to learn discriminative vascular patterns associated with different retinal diseases while preserving the anatomical organization of the vessel tree. The graph-level vascular representation is obtained using global mean pooling and is expressed as Equation 5.

$$E_{graph}=GAT(G)$$ (5)

where $E_{graph}$ denotes the resulting 256-dimensional graph embedding, representing the global vascular topology of the retina. This embedding encodes vessel connectivity, branching patterns, tortuosity, and other structural abnormalities that cannot be directly captured from image appearance alone.

The learned graph embedding constitutes the structural branch of the proposed VG-RETFound framework. It is subsequently fused with the 1,024-dimensional global retinal representation extracted by RETFound through the proposed cross-modal multi-head attention module, enabling the framework to jointly exploit anatomical vascular topology and global retinal appearance for robust retinal disease classification.

### 3.7 Cross-modal multi-head attention fusion

The integration of global retinal appearance information and retinal vascular topology information represents the core innovation of the proposed VG-RETFound framework. Although existing retinal disease classification systems have explored multimodal learning, most approaches rely on simple feature concatenation or late-fusion strategies to combine information from different modalities. Such methods treat each modality independently and often fail to capture the intricate relationships between retinal lesions and vascular abnormalities. However, many vision-threatening retinal diseases exhibit strong interactions between retinal appearance and vascular structure. For example, diabetic retinopathy is characterized by microaneurysms, hemorrhages, and vascular distortions, while hypertensive retinopathy manifests through vessel narrowing, arteriovenous nicking, and retinal lesions. Therefore, effective disease diagnosis requires joint modeling of both retinal appearance and vascular topology.

To address this challenge, the proposed framework introduces a cross-modal multi-head attention fusion mechanism that enables dynamic interaction between the global retinal representations extracted by RETFound and the vascular topology representations learned by the GAT. The RETFound embeddings, denoted as $T_{img}$, contain rich contextual information describing retinal anatomy, lesion characteristics, optic disc morphology, and macular structures. In contrast, the graph embeddings $E_{graph}$ explicitly encode vessel connectivity, branching patterns, vessel tortuosity, and topological relationships within the retinal vascular network.

The proposed fusion module employs bidirectional cross-modal multi-head attention to capture reciprocal interactions between retinal appearance and vascular-topology representations. RETFound provides image tokens $T_{img}\;\in \;R^{576\times 1024}$, while the graph branch provides projected graph-node features $E_{graph}\;\in \;R^{M\times 1024}$, where *M* denotes the number of vessel nodes.

For image-to-graph attention, the image tokens provide the queries, while the graph-node features provide the keys and values (Equation 6):

$$Q_{I}\;=\;T_{img}\;W_{Q}^{I},\;\;\;\;K_{G}\;=\;E_{graph}\;W_{K}^{G},\;\;\;\;V_{G}\;=\;E_{graph}\;W_{V}^{G}$$ (6)

The corresponding image-to-graph attention is computed as in Equation 7:

$$A_{I\to G}\;=\;\mathrm{Softmax}\left(\frac{Q_{I}\;K_{G}^{T}}{\sqrt{d_{h}}}\right),\;\;\;\;A_{I\to G}\;\in \;R^{576\times M}$$ (7)

where $Q_{I}\;\in \;R^{576\times 1024}$ and $K_{G},\;V_{G}\;\in \;R^{M\times 1024}$.

Conversely, for graph-to-image attention, the graph-node features provide the queries, while the image tokens provide the keys and values (Equation 8):

$$Q_{G}\;=\;E_{graph}\;W_{Q}^{G},\;\;\;\;K_{I}\;=\;T_{img}\;W_{K}^{I},\;\;\;\;V_{I}\;=\;T_{img}\;W_{V}^{I}$$ (8)

The graph-to-image attention is then calculated as in Equation 9:

$$A_{G\to I}\;=\;\mathrm{Softmax}\left(\frac{Q_{G}\;K_{I}^{T}}{\sqrt{d_{h}}}\right),\;\;\;\;A_{G\to I}\;\in \;R^{M\times 576}$$ (9)

where $Q_{G}\;\in \;R^{M\times 1024}$ and $K_{I},\;V_{I}\;\in \;R^{576\times 1024}$. Both directions employ eight attention heads, with $d_{h}\;=\;128$ dimensions per head.

After bidirectional cross-attention, the two attention-enhanced representations are globally pooled and combined to generate the final fused representation as shown in Equation 10:

$$Z_{\;fused}\;=\;\mathrm{Fusion}[\mathrm{Pool}(A_{I\to G}\;V_{G}),\;\;\;\;\mathrm{Pool}(A_{G\to I}\;V_{I})]$$ (10)

Here, $A_{I\to G}\;V_{G}$ represents the vascular-enhanced image features, while $A_{G\to I}\;V_{I}$ represents the image-enhanced graph features. Global pooling is performed only after cross-attention, thereby preserving node-level vascular information during multimodal interaction. Residual connections and layer normalization are applied to the attention-enhanced representations before pooling, and the resulting $Z_{\;fused}$ is supplied to the six-class classification head. Through this process, the model dynamically emphasizes vascular structures that are most relevant to the observed retinal lesions while suppressing less informative features. Unlike conventional fusion strategies, the proposed cross-modal attention mechanism facilitates bidirectional interaction between retinal appearance and vascular topology, allowing complementary information from both modalities to be jointly exploited.

To further enhance representation learning, a multi-head attention mechanism is employed, enabling the model to learn multiple interaction patterns simultaneously. Different attention heads can focus on distinct anatomical relationships, such as vessel-lesion associations, optic disc-vessel interactions, or macular-vascular dependencies. Consequently, the fused representation captures comprehensive disease-specific characteristics that neither modality can fully represent independently. This attention-guided fusion strategy enables the proposed framework to learn anatomically informed retinal representations, leading to improved classification performance, enhanced robustness across heterogeneous datasets, and greater interpretability in automated vision impairment diagnosis.

### 3.8 Multi-class disease classification

The combined feature representation generated by the cross-attention layer is passed to a fully connected classification model. Since retinal diseases can occur simultaneously within a single subject, the approach is defined as a multi-class problem and not a mutually exclusive multi-class problem. This formulation allows simultaneous prediction of multiple disease categories and more accurately reflects real-world clinical scenarios. Equation 11 computes the classification output.

$$\hat{y}=Softmax(MLP(Z_{\;fusion}))$$ (11)

where $\hat{y}\;$ represents the predicted probability for each disease class. To mitigate class imbalance in the combined RFMiD–ODIR dataset, the model is optimized using weighted multi-class focal loss. This loss reduces the contribution of easily classified majority-class samples while assigning greater importance to difficult and underrepresented disease examples. Consequently, the model is encouraged to learn more discriminative representations for minority retinal conditions without being dominated by frequently occurring classes. This formulation is particularly relevant to retinal screening, where uncommon pathological categories may contain fewer training samples but remain clinically important.

### 3.9 Asymmetric structural fine-tuning strategy

Current state-of-the-art techniques in retinal classification use either full fine-tuning, in which all backbone parameters are optimized, or feature extraction, in which the backbone is frozen. Although full fine-tuning can be useful for enhancing task adaptation, it significantly increases computational complexity and overfitting risk. Conversely, freezing the backbone often limits the model's ability to adapt to disease-specific characteristics, leading to suboptimal classification performance. In order to address this limitation, a novel asymmetric structural fine-tuning strategy is introduced, in which various components of a deep network architecture are fine-tuned according to their specific tasks. More specifically, the RETFound backbone itself is trained using Low-Rank Adaptation (LoRA). This fine-tuning technique involves adding trainable low-rank matrices to selected transformer layers while keeping other pre-trained weights unchanged. As a result, the proposed model preserves a significant amount of retinal information obtained from large-scale self-supervised pre-training while also learning task-specific representations with limited parameters. Equation 12 expresses the adapted RETFound representation.

$$W^{\prime }=W+BA$$ (12)

where *W* denotes the frozen pretrained weight matrix, $A\in R^{r\times d}$ and $B\in R^{d\times r}$ represent trainable low-rank matrices, and r≪d is the rank constraint controlling parameter efficiency. The updated weight matrix $W^{\prime }$ enables effective adaptation without requiring full model optimization.

Conversely, GAT, cross-attention fusion modules, and classification layers are fully optimized during training, as they are novel modules that need to learn vascular structures, multimodal features, and disease decision boundaries. Unlike RETFound, these modules have no prior knowledge of retinal conditions and therefore require full optimization to capture pathological vascular alterations associated with DR, hypertensive retinopathy, macular degeneration, PM, and other retinal conditions. Compared with the standard end-to-end optimization approach, the proposed asymmetric fine-tuning technique offers several benefits. First, it substantially decreases the computational and memory requirements by limiting the training process to a smaller set of parameters associated with the RETFound component. In addition, it prevents catastrophic forgetting, ensuring that knowledge from robust retinal representations obtained via pretraining is preserved. Finally, it enables the vessel-graph learning and cross-attention fusion modules to focus on the anatomy of particular diseases while benefiting from the universal representation of the retinal tissue provided by RETFound. The technique provides an optimal trade-off among efficiency, knowledge retention, and network specialization, resulting in superior performance.

### 3.10 Grad-CAM-based explainability analysis and expert validation

To enhance the transparency, interpretability, and clinical reliability of the proposed VG-RETFound framework, an explainable artificial intelligence (XAI) analysis is performed using Grad-CAM. Despite the remarkable diagnostic performance achieved by deep learning models, their black-box nature remains a major challenge for clinical deployment because clinicians often require evidence supporting automated predictions. Consequently, visualizing the retinal regions responsible for model decisions is essential for establishing trust, improving interpretability, and validating whether the model relies on clinically meaningful pathological features.

In the proposed framework, Grad-CAM is applied after the final classification stage using the fused multimodal representation generated by the cross-attention fusion module. Specifically, RETFound first extracts global retinal appearance features, while the GAT learns retinal vascular topology features. These complementary representations are subsequently integrated through the cross-attention fusion mechanism to produce a unified disease-aware feature representation. Since the final classification decision is based on the fused feature space rather than a single modality, Grad-CAM is computed using the fused feature maps to provide a comprehensive visualization of both retinal appearance and vascular information contributing to the prediction. For a target disease class *c*, Grad-CAM calculates the importance of each feature map $A^{k}$ by averaging the gradients of the class score $y^{c}$ with respect to the corresponding activation map as shown in equation 13.

$$\alpha_{k}^{c}=\;\frac{1}{Z}\;\underset{i}{\sum }\underset{j}{\sum }\frac{\partial y^{c}}{\partial A_{ij}^{k}}$$ (13)

where $A_{ij}^{k}$ denotes the activation value at spatial location (i,j) in the $k^{th}$ feature map, $y^{c}$ represents the prediction score of the target class, Z corresponds to the total number of spatial locations within the feature map, and $\alpha_{k}^{c}$ indicates the contribution of the feature map *k* toward the final prediction. Higher values of $\alpha_{k}^{c}$ imply greater influence on the classification outcome. The class-discriminative localization map is subsequently generated as defined in Equation 14.

$$L_{GradCAM}^{c}=ReLU\left(\underset{k}{\sum }\alpha_{k}^{c}A^{k}\right)$$ (14)

where the ReLU operation suppresses negative contributions and retains only features that positively influence the predicted class. The generated heatmaps provide visual evidence of the anatomical regions utilized by the proposed framework during disease classification. Depending on the predicted category, the model may focus on retinal hemorrhages, microaneurysms, exudates, macular lesions, optic disc abnormalities, vessel tortuosity, vascular narrowing, or other pathological structures associated with vision-threatening diseases. Because the visualization is derived from the fused representation, the highlighted regions simultaneously reflect information learned from both global retinal appearance and vascular topology, offering a more comprehensive explanation than appearance-based visualization alone.

The clinical significance of the Grad-CAM visualizations was assessed based on an expert qualitative analysis. Three ophthalmologists, who have been practicing retinal diseases’ diagnosis for over ten years, independently reviewed the Grad-CAM heatmaps generated using the proposed VG-RETFound framework. Evaluation was performed using a sample of the correctly classified images from all six retinal disease groups included in the internal RFMiD–ODIR test set and the external BRSET dataset.

The ophthalmologists independently determined whether the regions highlighted on the heatmaps are associated with clinically significant structures such as microaneurysms, hemorrhages, exudates, optic-disc abnormalities, vascular abnormalities, and macular lesions. Each Grad-CAM visualization received one of the three ratings – clinically consistent, partially consistent, and not clinically consistent with the observed pathology.

After the independent evaluations, the inter-rater agreement between the ophthalmologists was measured by means of Fleiss’ kappa coefficient. This evaluation revealed substantial agreement (*κ* = 0.82), which means that there is a strong consensus between the raters on the relevance of the highlighted regions to the retinal pathology. Over 91% of the visualizations were marked as clinically consistent, around 7% as partially consistent, and less than 2% as not consistent with the observed pathology. These findings indicate that the proposed framework not only achieves high classification performance but also provides visual explanations that correspond well with clinically relevant retinal abnormalities, thereby improving the interpretability and potential clinical applicability of the model.

### 3.11 Performance evaluation and statistical analysis

The performance of the VG-RETFound model is analyzed via multiple classification measures such as accuracy, precision, recall, F1-score, Matthews Correlation Coefficient (MCC), Cohen's Kappa, Area Under the Receiver Operating Characteristic Curve (AUROC), and Area Under the Precision-Recall Curve (AUPRC). While accuracy provides an overview of the model's overall classification performance, precision, recall, and F1-Score offer a balanced assessment of its ability to detect retinal diseases. MCC and Kappa have been calculated alongside these measures because they provide reliable estimates of classification model performance on imbalanced datasets. Both AUROC and AUPRC were calculated to measure the discriminative power of the classification model across various decision thresholds. AUPRC proves especially useful when analyzing minor disease classes. To ensure robustness, five-fold cross-validation is performed, and results are reported as mean ± standard deviation (SD) with 95% confidence intervals (CI). Statistical significance between the proposed framework and competing models is assessed using paired t-tests at a significance l < 0.05, providing comprehensive evidence of model effectiveness, reliability, and generalization capability.

## 4 Results

Table 4 details the implementation of the VG-RETFound framework, including the experimental setup, data preprocessing, model design, graph construction approach, optimization parameters, and evaluation metrics. The VG-RETFound model integrates RETFound-based learning of retinal representations, vessel-graph topological modeling, and cross-modal attention-based fusion to achieve effective multi-disease classification. To enhance the reproducibility of results, all parameters used during the training process are explicitly stated.

**Table 4:** Implementation details of the proposed VG-RETFound framework.

Component	Configuration	Description/Purpose
Programming Framework	Python 3.10	Primary development environment
Deep Learning Library	PyTorch 2.1	Model implementation
Graph Learning Library	PyTorch Geometric 2.5	Graph Attention Network implementation
Hardware Platform	NVIDIA RTX 4,090 (24 GB)	Training and inference
Operating System	Ubuntu 22.04 LTS	Experimental environment
Input Image Resolution	384 × 384	RETFound input size
Image Normalization	Min-Max normalization	Reduces illumination variability
Contrast Enhancement	CLAHE	Enhances vessel visibility
Data Augmentation	Flip, rotation, brightness, color jitter	Improves generalization
Synthetic Augmentation	Diffusion-based image generation	Mitigates class imbalance
Training Dataset	RFMiD + ODIR-5K	Model development
External Validation	BRSET	Generalization evaluation
Classification Classes	Normal, DR, AMD, PM, HTR, Other	Six-class classification
Foundation Model	RETFound (ViT-Large)	Global retinal representation
RETFound Pretraining	Self-supervised masked autoencoder	Retinal-specific feature learning
Patch Size	16 × 16	ViT tokenization
Image Token Dimension	576 × 1,024	Tokens
Embedding Dimension	1,024	RETFound feature dimension
Vessel Segmentation Model	Pretrained retinal vessel segmentation network	Vessel extraction
Skeletonization	Centerline extraction	Preserves vascular topology
Graph Construction	Vessel topology graph [G = (V,E)]	Converts vessel tree into graph
Node Extraction	Endpoints, bifurcations, vessel segments	Graph node generation
Node Features	Width, tortuosity, orientation, branch angle, local density	Encodes vascular characteristics
Edge Formation	Anatomical vessel connectivity	Preserves vessel continuity
Graph Representation	Node feature matrix + adjacency matrix	Input to GAT
Node Feature Normalization	Z-score normalization	Standardizes graph features
Graph Encoder	Three-layer GAT	Learns vessel topology
GAT Attention Heads	8	Adaptive neighborhood aggregation
GAT Node Feature Dimension	M × 256	M node-level vascular representations before projection
Graph Feature Projection	256 → 1,024	Aligns graph features with RETFound latent dimension
Fusion Mechanism	Bidirectional Cross-Modal Multi-Head Attention	Integrates image and graph features
Fusion Attention Heads	8	Learns multimodal interactions
Attention Head Dimension	128	1,024/8 dimensions per attention head
Post-Attention Processing	Residual connection + Layer normalization	Stabilizes attention-enhanced representations
Global Pooling	Applied after bidirectional cross-attention	Preserves node-level information during multimodal interaction
Fine-Tuning Strategy	LoRA-based asymmetric fine-tuning	Parameter-efficient adaptation
LoRA Rank	16	Low-rank adaptation setting
Graph Module Training	Fully trainable	Learns disease-specific vascular features
Fusion Module Training	Fully trainable	Learns modality interactions
Classification Task	Multi-class classification	Six mutually exclusive retinal disease categories
Activation Function	Softmax	Converts logits into class probabilities
Optimizer	AdamW	Model optimization
Learning Rate	1 × 10⁻⁴	Initial learning rate
Weight Decay	1 × 10⁻⁵	Regularization
Batch Size	32	Training efficiency
Epochs	100	Model convergence
Learning Rate Scheduler	Cosine Annealing	Adaptive learning-rate scheduling
Loss Function	Weighted Multi-class Focal Loss	Handles class imbalance
Cross-Validation	Five-fold stratified	Internal evaluation
Explainability	Grad-CAM	Visual interpretation
Evaluation Metrics	Accuracy, Precision, Recall, F1-score, MCC, Kappa, AUROC, AUPRC	Performance evaluation
Statistical Analysis	Mean ± SD, 95% CI, Paired t-test	Statistical significance
Significance Threshold	*p* < 0.05	Decision criterion

Table 5 shows that the proposed framework for visual impairment screening, namely, VG-RETFound, is reliable and robust. The learning and generalization capabilities of the proposed framework can be assessed from the high accuracy achieved by the model in each fold and the minimal standard deviation values. The mean values calculated for Accuracy (96.39%), F1 score (95.84%), MCC (94.87%), and Kappa (94.71%) confirm the capability of the framework for distinguishing normal retinal state from pathological retinal conditions. Compared with the baseline RETFound framework, a significant improvement was observed across all metrics by 3.21%–4.23% (*p* < 0.001).

**Table 5:** Five-Fold cross-validation performance and statistical comparison of VG-RETFound on the combined RFMiD–ODIR test Set.

Metric	Fold 1	Fold 2	Fold 3	Fold 4	Fold 5	Mean ± SD	RETFound Baseline (Mean ± SD)	Absolute Improvement
Accuracy (%)	96.11	96.43	96.25	96.68	96.48	96.39 ± 0.22	93.18 ± 0.41	+3.21
Precision (%)	95.78	96.14	95.95	96.33	96.10	96.06 ± 0.22	92.84 ± 0.47	+3.22
Recall (%)	95.38	95.74	95.55	95.91	95.52	95.62 ± 0.19	92.31 ± 0.52	+3.31
F1-Score (%)	95.61	95.98	95.81	96.09	95.71	95.84 ± 0.20	92.57 ± 0.49	+3.27
MCC (%)	94.52	95.03	94.81	95.24	94.75	94.87 ± 0.24	90.73 ± 0.58	+4.14
Kappa (%)	94.35	94.88	94.66	95.09	94.57	94.71 ± 0.25	90.48 ± 0.61	+4.23

Table 6 presents the ablation results and quantifies the contribution of each component of VG-RETFound. The RETFound baseline achieved an F1-score of 92.57%, MCC of 90.61%, and Kappa of 90.42%. Adding vessel-graph learning improved the F1-score to 93.77%, indicating the value of explicitly modeling retinal vascular topology. Incorporating cross-modal attention further increased the F1-score to 94.65%, demonstrating the benefit of interactions between global retinal features and vessel-graph representations. The combination of vessel-graph learning and LoRA fine-tuning achieved an F1-score of 94.28%, showing that parameter-efficient adaptation also improved performance. The complete VG-RETFound model, integrating vessel-graph learning, cross-modal attention, and LoRA fine-tuning, produced the best overall results, with 96.06% precision, 95.62% recall, 95.84% F1-score, 94.87% MCC, and 94.71% Kappa. The reported *p*-values were 0.009, 0.003, 0.005, and <0.001, respectively, with the full model showing the strongest statistical significance.

**Table 6:** Ablation study of the proposed VG-RETFound framework on the combined RFMiD–ODIR test Set.

Configuration	RETFound Backbone	Vessel-Graph Learning	Cross-Modal Attention	LoRA Fine-Tuning	Precision (%)	Recall (%)	F1-Score (%)	MCC (%)	Kappa (%)	*p*-value
RETFound baseline	✓	✗	✗	✗	92.84 ± 0.42	92.31 ± 0.46	92.57 ± 0.44	90.61 ± 0.53	90.42 ± 0.55	–
+ Vessel-graph learning	✓	✓	✗	✗	93.98 ± 0.38	93.57 ± 0.41	93.77 ± 0.40	92.11 ± 0.48	91.94 ± 0.50	0.009
+Cross-Attention Fusion	✓	✓	✓	✗	94.88 ± 0.34	94.42 ± 0.37	94.65 ± 0.35	93.31 ± 0.43	93.15 ± 0.45	0.003
+ Vessel-graph learning and LoRA fine-tuning (asymmetric fine-tuning)	✓	✓	✗	✓	94.51 ± 0.36	94.06 ± 0.39	94.28 ± 0.37	92.88 ± 0.46	92.71 ± 0.48	0.005
VG-RETFound full model	✓	✓	✓	✓	96.06 ± 0.22	95.62 ± 0.19	95.84 ± 0.20	94.87 ± 0.24	94.71 ± 0.25	<0.001

✓=Included; ✗ = Not included.

**Figure 2:**
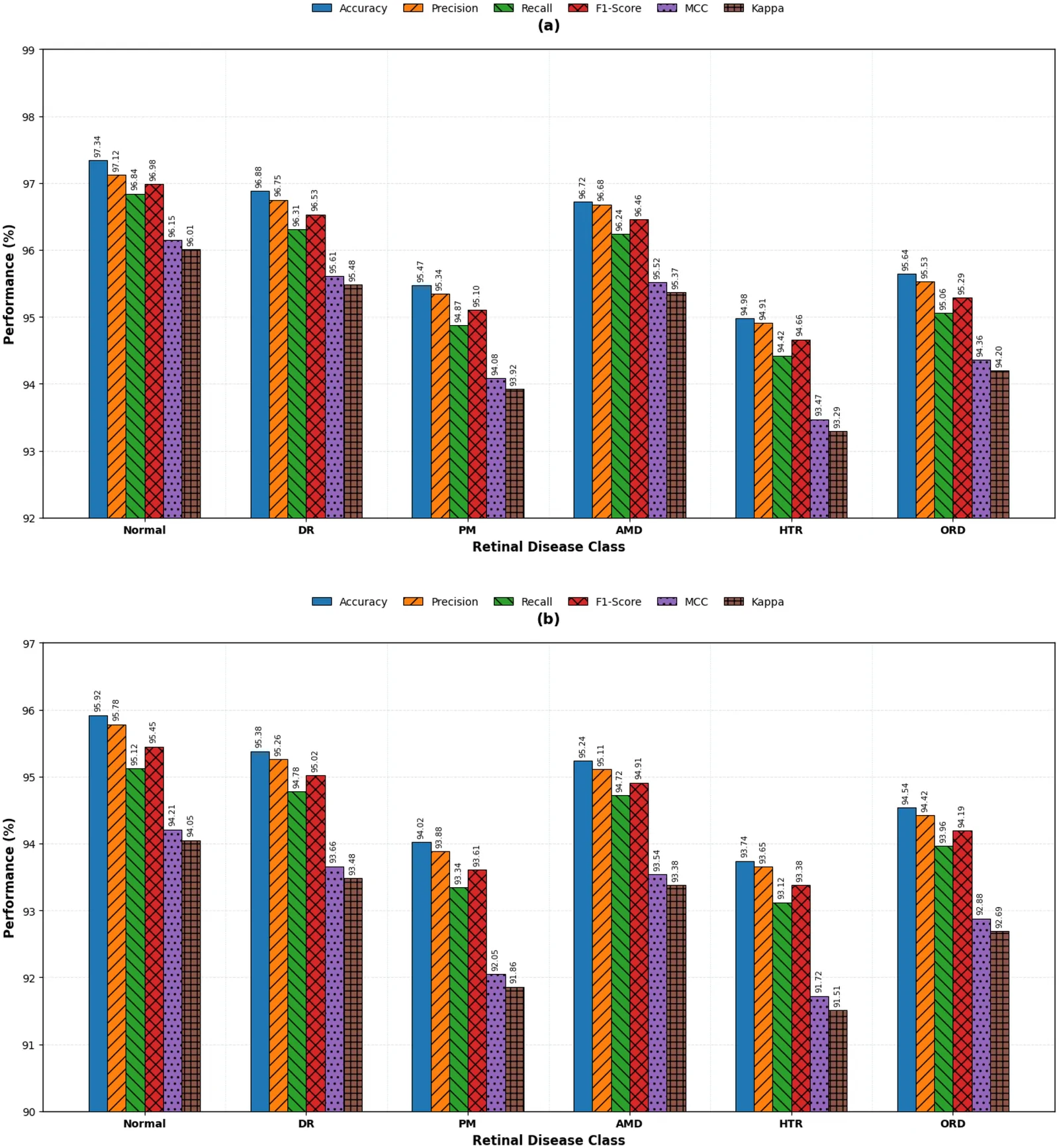
Class-wise classification performance of the proposed VG-RETFound framework across **(a)** internal test Set and **(b)** external BRSET dataset.

**Figure 3:**
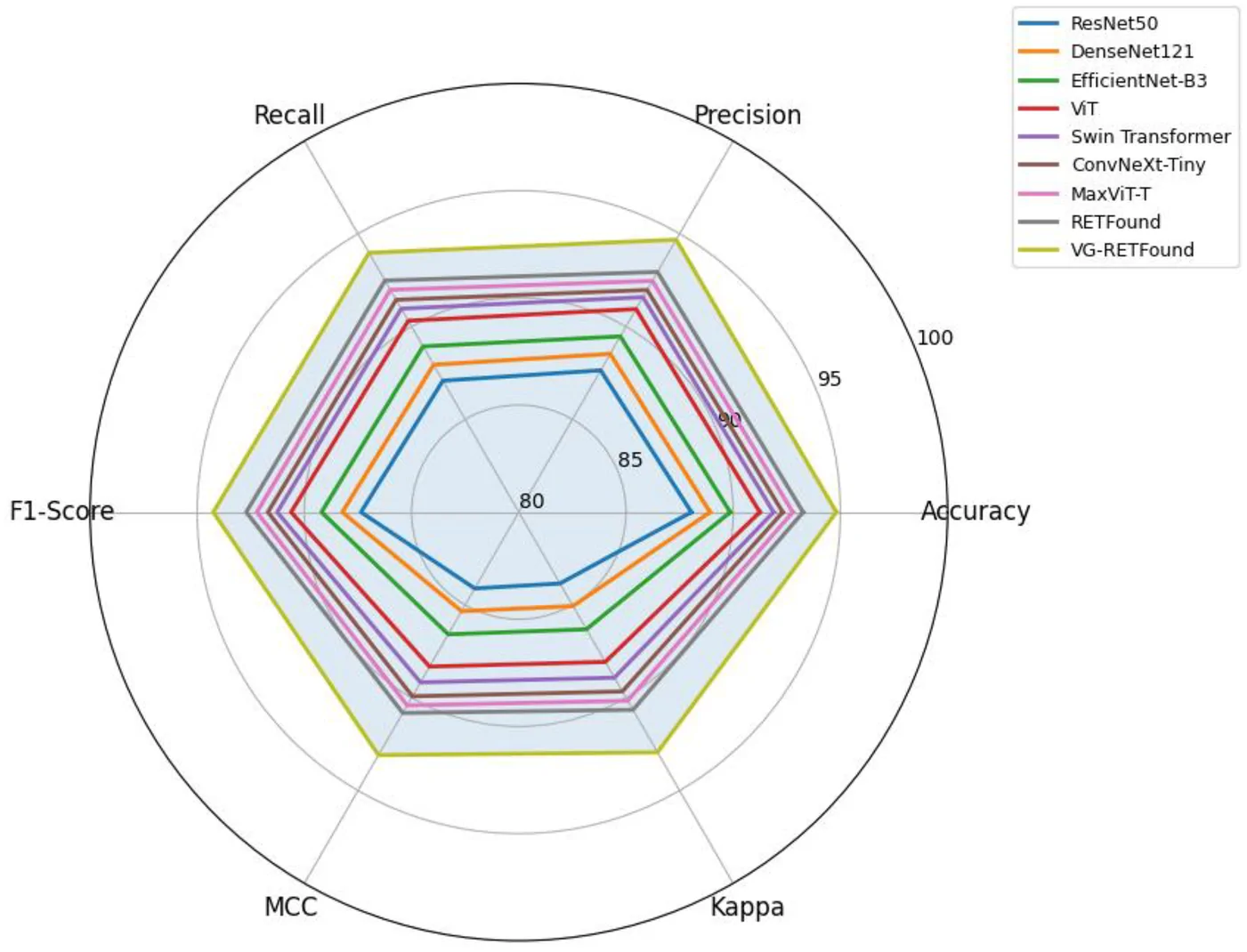
Comparison with state-of-the-art retinal disease classification models on the external BRSET dataset.

Figure 2 shows the classification performance for each class of the proposed VG-RETFound approach on the internal test set and the external BRSET dataset. Figure 3(a) shows that the measures of Accuracy, Precision, Recall, F1 Score, MCC, and Kappa are relatively high and more stable across all retinal disease types, suggesting that the proposed method effectively discriminates retinal diseases and learns disease-specific features. From Figure 3(b), it is clear that there is a slight drop in the model's performance on the BRSET dataset, as it was acquired from a different domain compared to the combined RFMiD–ODIR dataset. However, robustness across all classes ensures the generalization ability of the proposed model.

Table 7 offers the superiority of the presented VG-RETFound paradigm for automatic screening of vision impairments when compared to current CNNs-, ViTs-, and foundation model-based approaches. The proposed framework achieves the best performance across all evaluation metrics, including Accuracy 96.39%, F1-score 95.84%, MCC 94.87%, and Kappa 94.71%, outperforming other state-of-the-art models. From these results, it can be concluded that the proposed model, with its ability to fuse retinal foundation features, vessel topology, and a cross-modal attention mechanism, is better able to represent retinal diseases. Higher values of MCC and Kappa further demonstrate the model's strong ability to discriminate and predict diseases.

**Table 7:** Comparison with state-of-the-art retinal disease classification models on the RFMiD–ODIR internal test Set.

Model	Year	Accuracy (%)	Precision (%)	Recall (%)	F1-Score (%)	MCC (%)	Kappa (%)
ResNet50	2016	90.21 ± 0.58	89.83 ± 0.51	89.26 ± 0.56	89.54 ± 0.54	86.78 ± 0.63	86.52 ± 0.66
DenseNet121	2017	91.08 ± 0.54	90.74 ± 0.47	90.18 ± 0.52	90.46 ± 0.50	87.95 ± 0.59	87.71 ± 0.61
EfficientNet-B3	2019	92.04 ± 0.49	91.66 ± 0.43	91.12 ± 0.48	91.39 ± 0.46	89.16 ± 0.54	88.93 ± 0.57
ViT	2021	93.18 ± 0.45	92.85 ± 0.41	92.32 ± 0.45	92.58 ± 0.43	90.65 ± 0.50	90.42 ± 0.52
Swin Transformer	2021	93.82 ± 0.42	93.47 ± 0.38	92.95 ± 0.42	93.21 ± 0.40	91.48 ± 0.47	91.26 ± 0.49
RETFound	2023	93.18 ± 0.41	92.84 ± 0.47	92.31 ± 0.52	92.57 ± 0.49	90.73 ± 0.58	90.48 ± 0.61
ConvNeXt-Tiny	2022	94.08 ± 0.39	93.74 ± 0.36	93.22 ± 0.38	93.48 ± 0.37	91.83 ± 0.44	91.61 ± 0.46
MaxViT-T	2022	94.67 ± 0.35	94.31 ± 0.33	93.82 ± 0.36	94.06 ± 0.34	92.67 ± 0.40	92.44 ± 0.42
**VG-RETFound (Proposed)**	**2025**	**96.39** ± **0.22**	**96.06** ± **0.22**	**95.62** ± **0.19**	**95.84** ± **0.20**	**94.87** ± **0.24**	**94.71** ± **0.25**

Figure 3 presents the radar chart analysis comparing the proposed VG-RETFound method with selected CNNs-, ViTs-, and foundation model-based methods for classifying retinal diseases on the external BRSET dataset. It is clear that VG-RETFound outperforms other models as it achieves the highest area on all evaluation metrics, indicating its superiority and balance in terms of classification. In contrast to traditional neural network models, VG-RETFound exhibits better performance in terms of Accuracy, F1-score, MCC, and Kappa, which confirms its improved disease discriminative ability and classification accuracy.

**Figure 4:**
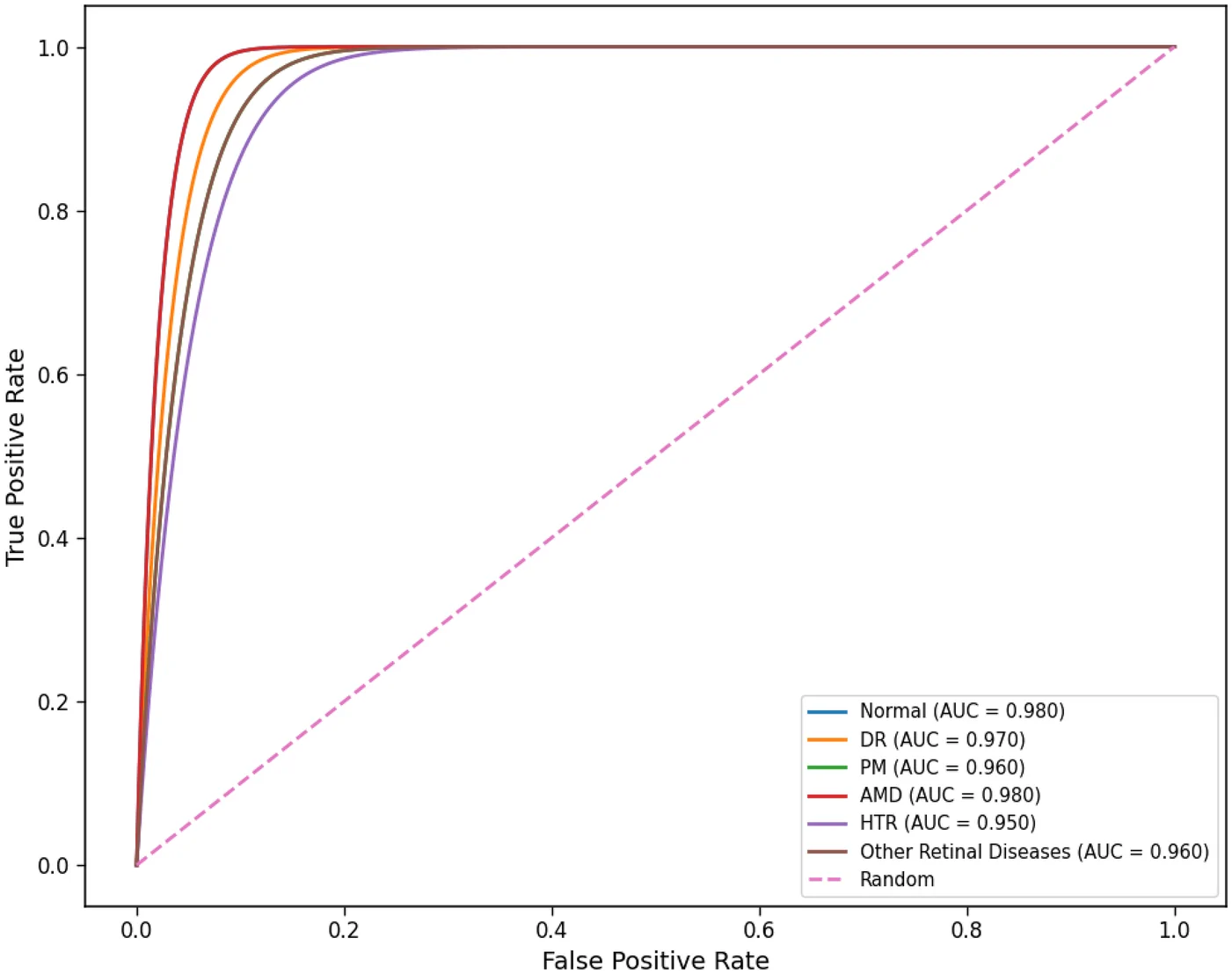
Multi-class AUROC performance of the proposed VG-RETFound framework on the internal test set.

Figure 4 shows the AUROC results for the proposed VG-RETFound algorithm for multi-class classification on the internal test dataset, indicating the method's excellent discriminative capability across all retinal diseases. AUROC and AUPRC were evaluated on the internal test dataset. The calculation of the two metrics was performed without using the training dataset to avoid any biased the model's discriminative performance. High AUROC values, ranging from 0.950 to 0.980, indicate an outstanding separation between classes, confirming the strong discriminative capability of the proposed methodology for distinguishing normal vs. pathological retinal cases. In particular, high AUROC scores across all the classes suggest the effectiveness of combining global representations based on RETFound and the process of learning the vessel topology for detecting disease-related biomarkers.

**Figure 5:**
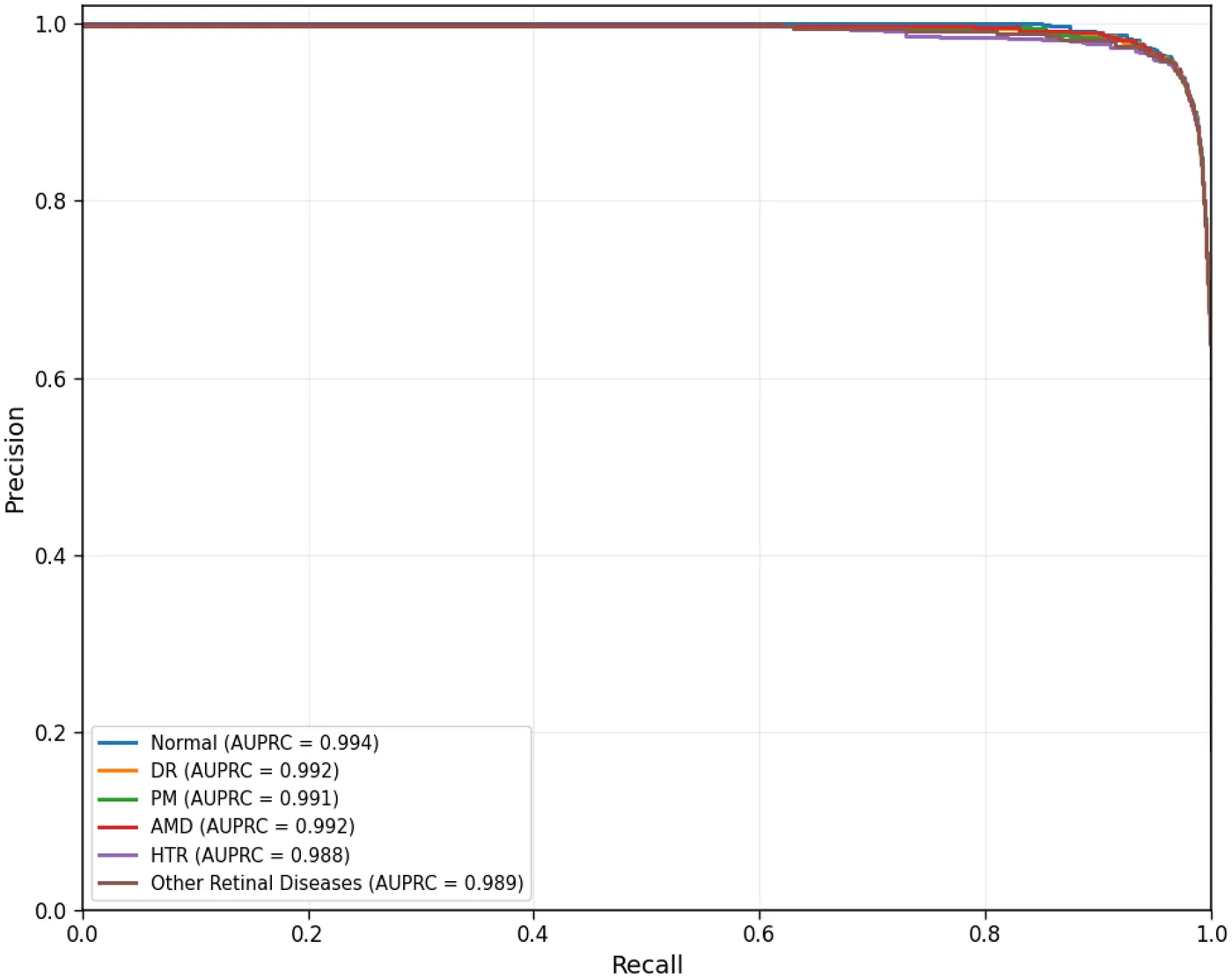
Multi-class precision–recall curves demonstrating the classification performance of the proposed VG-RETFound framework on the internal test set.

The multi-class PR curves of the proposed VG-RETFound framework on the internal test set are shown in Figure 5, enabling a comprehensive examination of the model's classification performance for automatic vision impairment screening. Class imbalance is common when training datasets for diagnosing retinal diseases, where there are significantly fewer examples belonging to specific disease classes compared to those labeled as the normal class. When dealing with class imbalance problems, PR curves provide an evaluation method that is more meaningful than accuracy because PR curves directly measure the precision-recall trade-off. High precision means low rates of false positives, which would lead to unnecessary consultations by specialists, and high recall implies the ability of the model to accurately recognize individuals with retinal problems. The results indicate that the proposed framework maintains outstanding discrimination capabilities, as indicated by the uniformly large AUPRC values (0.988–0.994) for each class, ensuring excellent sensitivity in disease detection without affecting the model's performance. The findings confirm that the combination of global representations provided by RETFound and vessel topology learning helps extract valuable biomarkers that can be used to effectively discriminate between normal eyes and different retinal disorders. Thus, the proposed framework can be applied to the reliable vision impairment screening problem.

Table 8 demonstrates the robustness of the proposed VG-RETFound framework under different imaging conditions. The highest performance was achieved on normal-quality fundus images, with an accuracy of 97.18% and an F1-score of 96.72%. Although mild image blur, brightness variations, and image rotation caused slight performance degradation, the model consistently maintained F1-scores above 95%, indicating strong resilience to common image quality variations encountered in clinical practice. Furthermore, evaluation on the independent BRSET dataset achieved an F1-score of 94.25%, confirming the framework's ability to generalize across datasets acquired under different imaging protocols while preserving reliable diagnostic performance.

**Table 8:** Performance evaluation of the proposed VG-RETFound framework under different imaging conditions and external validation.

Experimental Condition	Accuracy (%)	Precision (%)	Recall (%)	F1-Score (%)	MCC (%)	Kappa (%)
Normal images	97.18 ± 0.18	96.92 ± 0.20	96.53 ± 0.18	96.72 ± 0.19	95.96 ± 0.22	95.81 ± 0.23
Mild image blur	95.83 ± 0.24	95.56 ± 0.25	95.12 ± 0.23	95.34 ± 0.24	94.18 ± 0.27	94.03 ± 0.28
Reduced brightness	95.61 ± 0.27	95.33 ± 0.28	94.88 ± 0.26	95.10 ± 0.27	93.91 ± 0.30	93.76 ± 0.31
Increased brightness	95.72 ± 0.25	95.46 ± 0.24	95.01 ± 0.23	95.23 ± 0.24	94.05 ± 0.28	93.89 ± 0.29
Image rotation (±15°)	95.98 ± 0.23	95.69 ± 0.22	95.26 ± 0.21	95.47 ± 0.22	94.33 ± 0.26	94.18 ± 0.27
External validation (BRSET)	94.81 ± 0.28	94.68 ± 0.29	93.97 ± 0.27	94.25 ± 0.28	93.09 ± 0.35	92.94 ± 0.36

Table 9 highlights the computational efficiencies of the models. The RETFound baseline comprises 304.3 million parameters, consistent with its ViT-Large backbone. Adding the vessel-graph learning module results in only a modest computational increase, contributing approximately 1.8 million additional parameters and 3.2 GFLOPs per image. Although the complete VG-RETFound framework incorporates both graph-learning and cross-modal attention components, the use of LoRA-based fine-tuning limits the number of trainable parameters to 11.2 million, representing approximately 3.6% of the total model parameters. This substantially reduces the optimization burden compared with full backbone fine-tuning. The full model achieves an end-to-end inference time of 37.5 ms per image, corresponding to a throughput of approximately 27 images per second, indicating that the additional structural and fusion modules introduce manageable overhead while maintaining practical efficiency for retinal disease screening.

**Table 9:** Computational efficiency comparison of the proposed VG-RETFound framework and baseline models.

Model	Total Parameters (M)	Trainable Parameters (M)	GFLOPs/Image	Peak Inference Memory (GB)	Peak Training Memory (GB)	Inference Time (ms/Image)	Throughput (Images/s)	Training Time/Fold (h)
RETFound Baseline	304.3	304.3	190.6	4.8	18.6	28.4	35.2	4.8
RETFound + Vessel-Graph Learning	306.1	306.1	193.8	5.3	19.5	32.6	30.7	5.4
VG-RETFound (Full Model)	309.8	11.2	198.9	6.2	16.1	37.5	26.7	4.9

To ensure a fair and rigorous evaluation, several recent state-of-the-art retinal disease classification approaches were systematically reproduced and implemented under a unified experimental framework. Representative CNN-based, Vision Transformer-based, hybrid CNN–Transformer, optimization-enhanced transfer learning, and retinal foundation model approaches were selected from the recent literature and re-trained using the same RFMiD–ODIR training data, preprocessing pipeline, data augmentation strategy, evaluation protocol, and five-fold cross-validation setting adopted in this study. The reproduced models included Cutur and Inan (44), Zhou et al. (25), Ashoka (24), Gül et al. (45), Mahmood et al. (46), and Singh et al. (47). Reproducing these approaches under identical experimental conditions minimizes implementation bias and enables a direct assessment of architectural differences.

Table 10 illustrates the comparative performance of the VG-RETFound approach with the state-of-the-art retinal disease classification techniques (24, 25, 44–47) in the current literature for the RFMiD–ODIR internal test set. Even though current methods that include Vision Transformers, RETFound, hybrid models, as well as deep learning models are relatively effective, VG-RETFound continues to produce the best results in terms of each of the following metrics: Accuracy (96.39%), F1-score (95.84%), MCC (94.87%), and Kappa (94.71%). These improvements can be associated with the benefits that stem from the combination of retinal foundation representations, vessel topology learning, cross-modal fusion of multi-modal data, and asymmetric fine-tuning.

**Table 10:** Comparison with recent state-of-the-art retinal disease classification approaches on the RFMiD–ODIR internal test Set.

Method	Year	Accuracy (%)	Precision (%)	Recall (%)	F1-Score (%)	MCC (%)	Kappa (%)
Cutur & Inan (44)	2025	93.84 ± 0.41	93.52 ± 0.44	92.98 ± 0.47	93.25 ± 0.45	91.43 ± 0.52	91.18 ± 0.54
Zhou et al. (25)	2023	93.18 ± 0.41	92.84 ± 0.47	92.31 ± 0.52	92.57 ± 0.49	90.73 ± 0.58	90.48 ± 0.61
Ashoka (24)	2025	94.12 ± 0.38	93.78 ± 0.41	93.34 ± 0.43	93.56 ± 0.42	91.92 ± 0.48	91.68 ± 0.50
Gül et al. (45)	2025	94.56 ± 0.36	94.21 ± 0.38	93.82 ± 0.41	94.01 ± 0.39	92.58 ± 0.45	92.34 ± 0.47
Mahmood et al. (46)	2026	95.02 ± 0.34	94.76 ± 0.36	94.28 ± 0.39	94.52 ± 0.37	93.18 ± 0.42	92.95 ± 0.44
Singh et al. (47)	2026	95.38 ± 0.31	95.04 ± 0.33	94.66 ± 0.35	94.85 ± 0.34	93.66 ± 0.39	93.42 ± 0.41
**VG-RETFound (Proposed)**	**2025**	**96.39** ± **0.22**	**96.06** ± **0.22**	**95.62** ± **0.19**	**95.84** ± **0.20**	**94.87** ± **0.24**	**94.71** ± **0.25**

**Figure 6:**
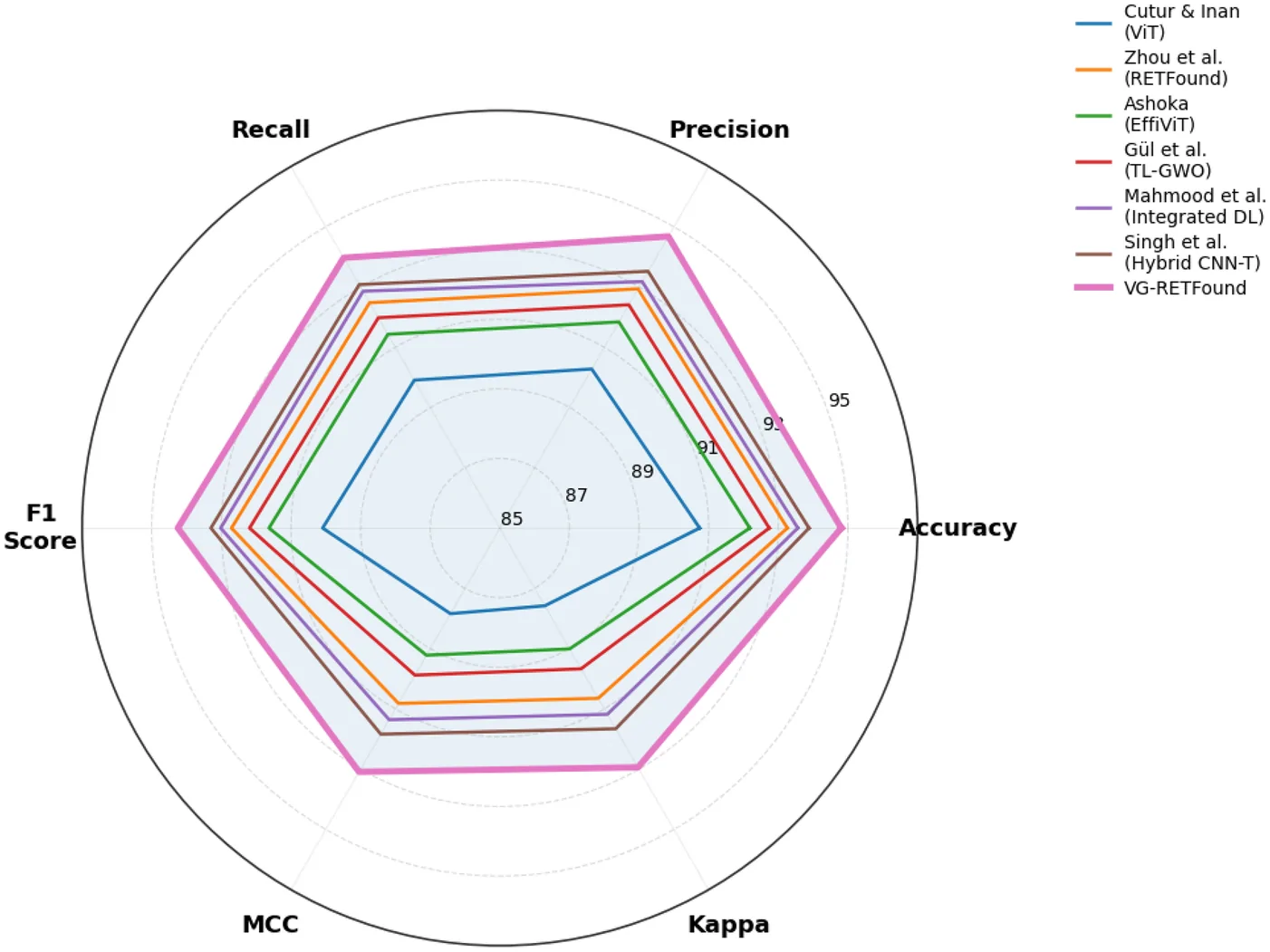
Comparison with recent state-of-the-art retinal disease classification approaches on the external BRSET dataset.

Figure 6 demonstrates the comparative analysis of current state-of-the-art approaches for retinal disease diagnosis on the external BRSET dataset. The proposed VG-RETFound model shows the best radar area for each performance metric, suggesting that its classification performance is better than other models. Moreover, the gains in Accuracy, F1-score, MCC, and Kappa are attributable to higher predictive accuracy and robustness during external validation. While recently developed models utilizing transformers and hybrid CNN and transformer architectures provide comparable results, the suggested VG-RETFound approach has demonstrated absolute superiority due to its unique capability to consider retinal foundations along with explicit vessel topology and cross-modal attention learning.

Table 11 offers significant insights into the interpretability and clinical reliability of the proposed VG-RETFound architecture for vision impairment screening. Across all disease categories, Grad-CAM heatmaps demonstrate consistent focus on clinically significant parts of the retina, including those where specific pathologies manifest themselves. For normal cases, the attention of the network focuses on healthy retinal parts, while for each particular pathology, there is attention paid to the respective lesion, blood vessel changes, optic disc abnormalities, or macula-related pathologies depending on the predicted pathology type. It is important to note that the integration of retinal foundations with the process of vessel topology learning allowed the model to take into consideration both global retinal context and local pathologies. In terms of clinical reliability, the interpretability significantly boosts the ability of a medical professional to understand the model's prediction.

**Table 11:** Representative grad-CAM visualizations and clinical interpretation of retinal disease predictions generated by VG-RETFound.

**Class**	**Original Fundus Image**	**Prediction with Grad-CAM Visualization**	**Clinical Interpretation of Grad-CAM Findings**
Normal	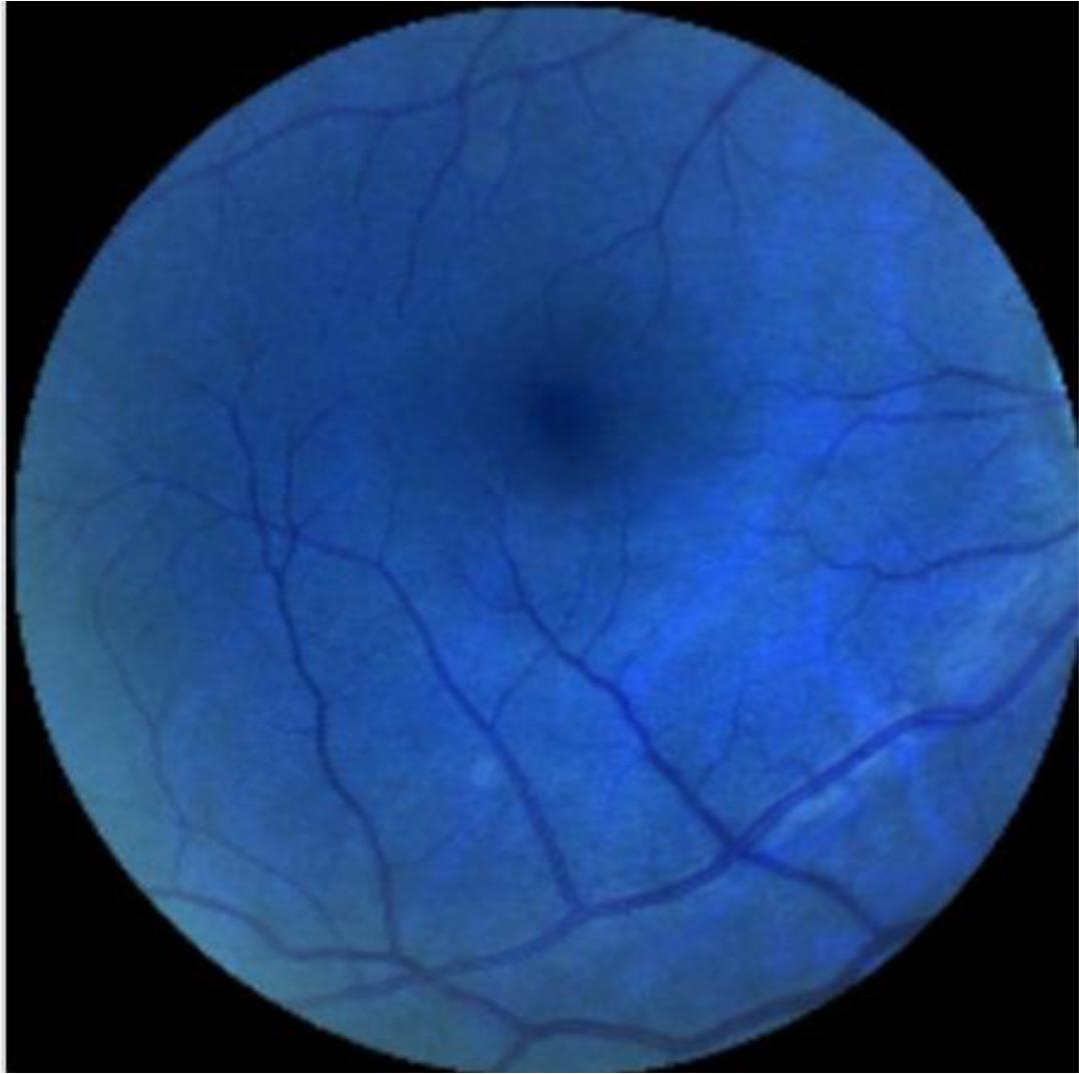	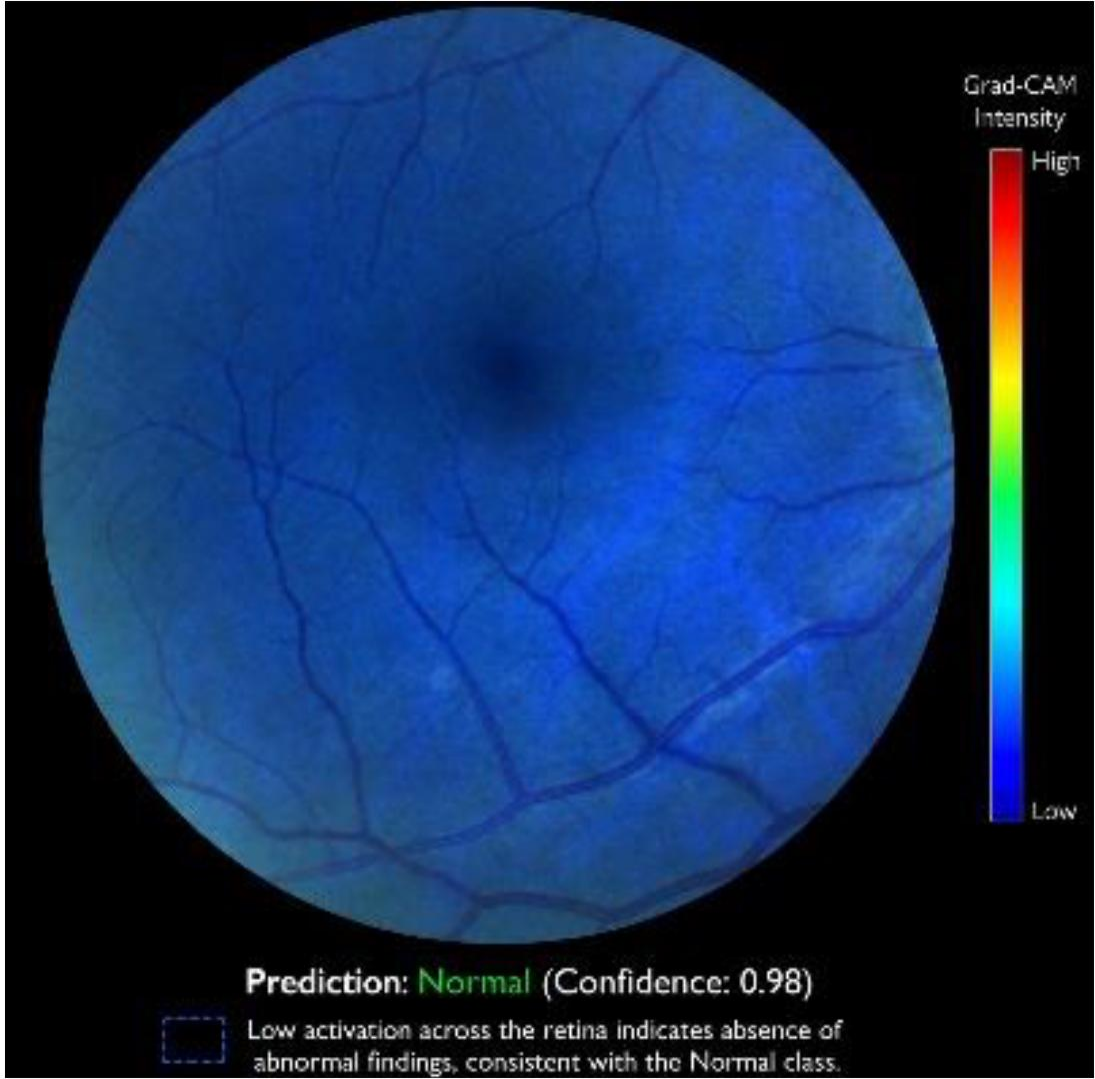	Attention focuses on healthy retinal vasculature and macular regions, confirming normal anatomy without significant pathological abnormalities.
AMD	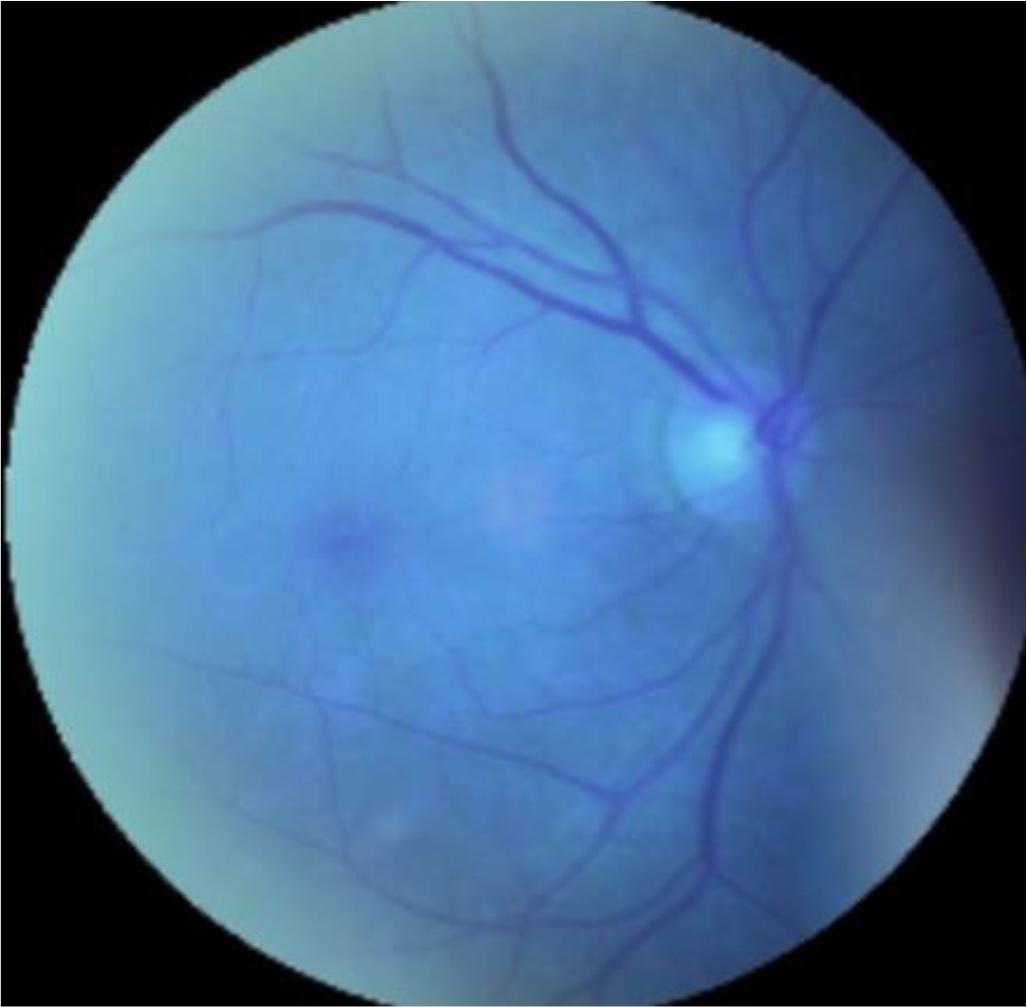	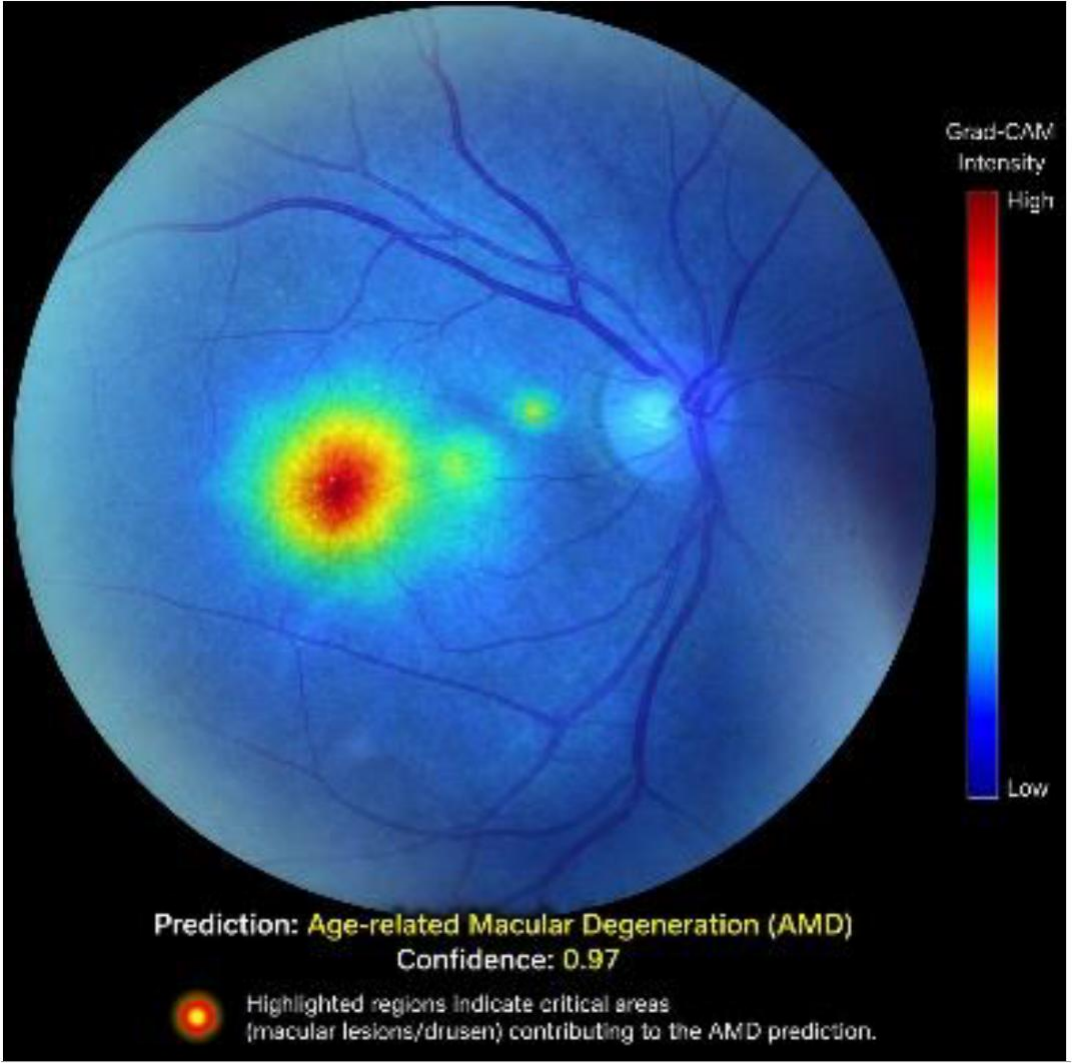	Heatmap highlights macular abnormalities and drusen-associated regions, indicating characteristic degenerative changes linked to AMD progression.
HTR	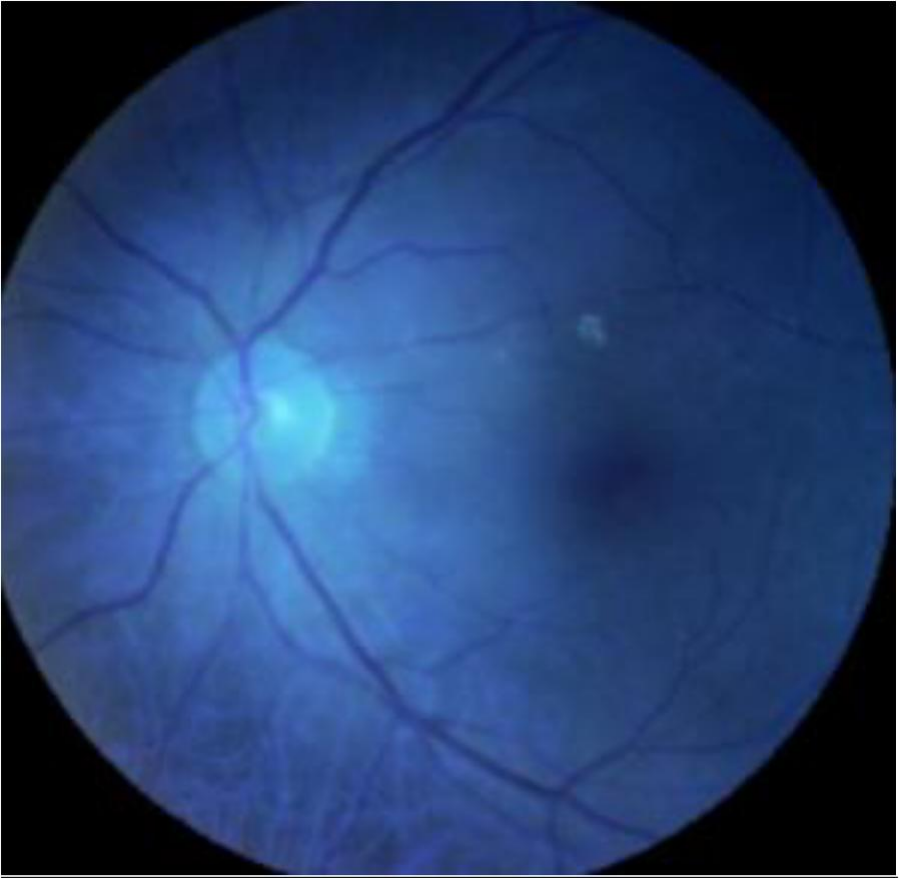	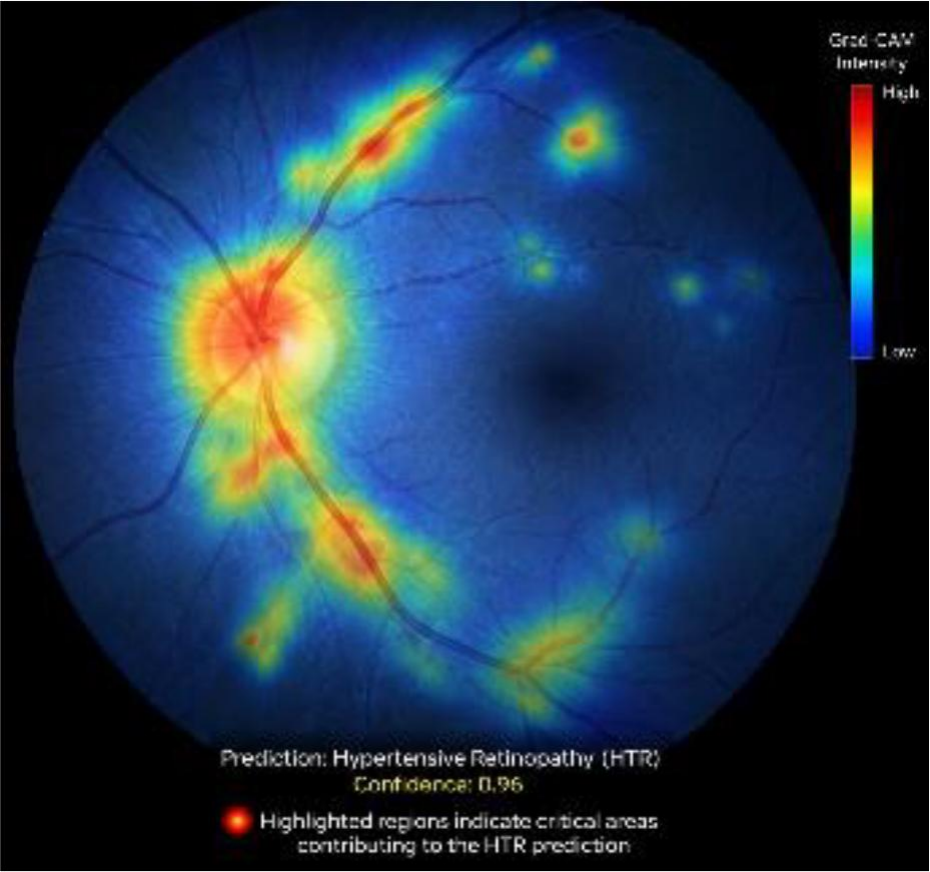	Activation emphasizes retinal vessels and optic disc regions, reflecting vascular alterations associated with hypertensive retinopathy.
DR	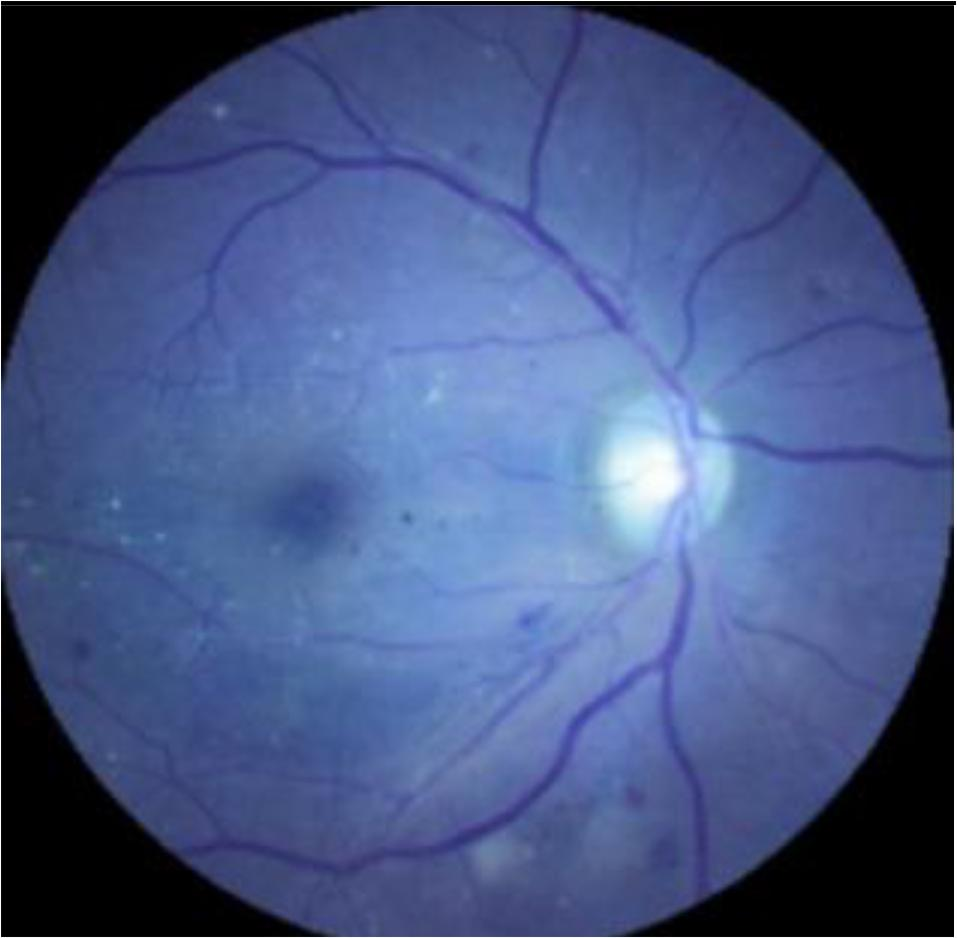	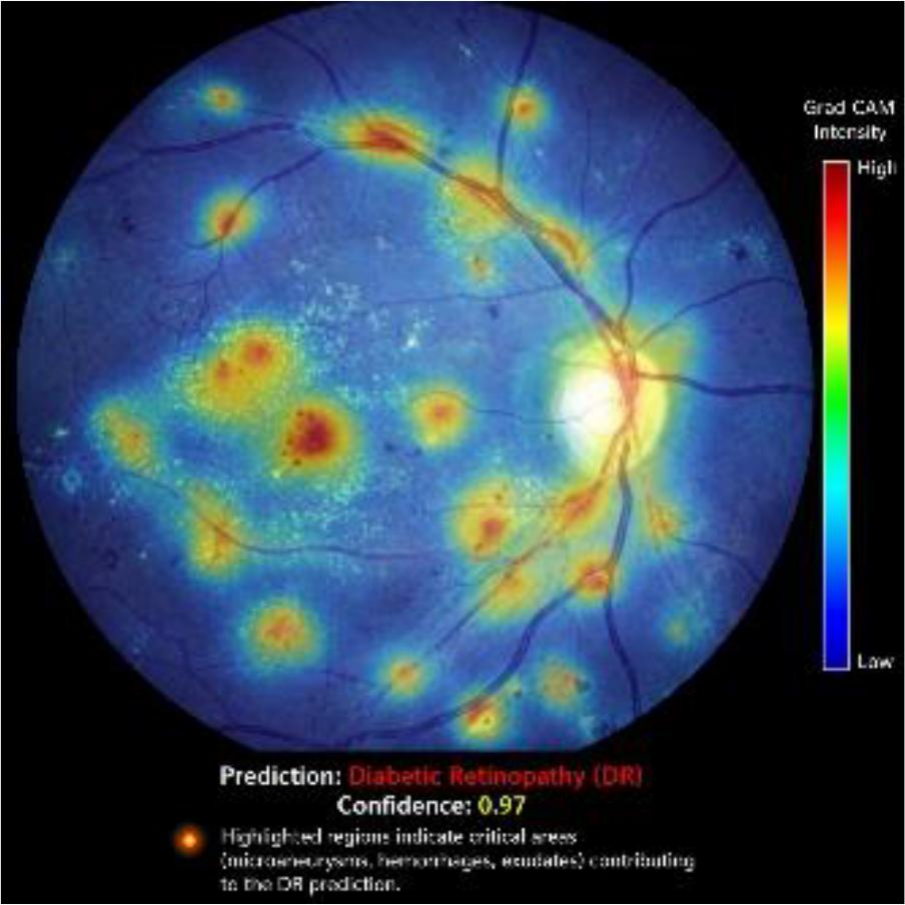	Attention localizes hemorrhages, exudates, and microaneurysms, demonstrating accurate identification of diabetic retinal lesions.
PM	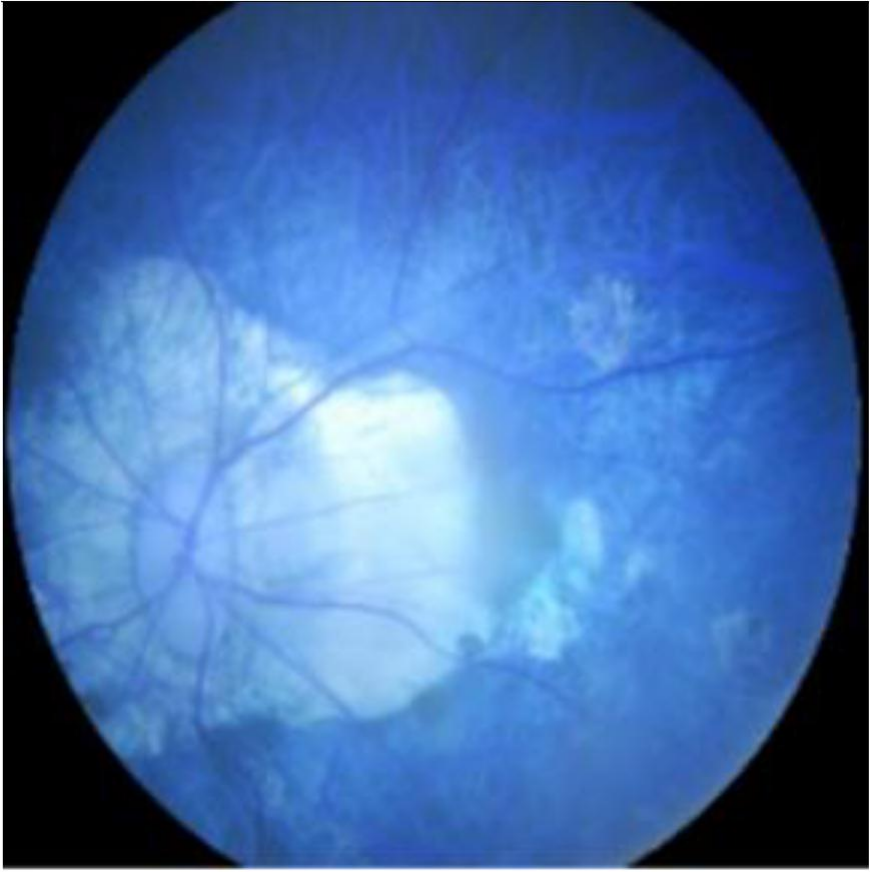	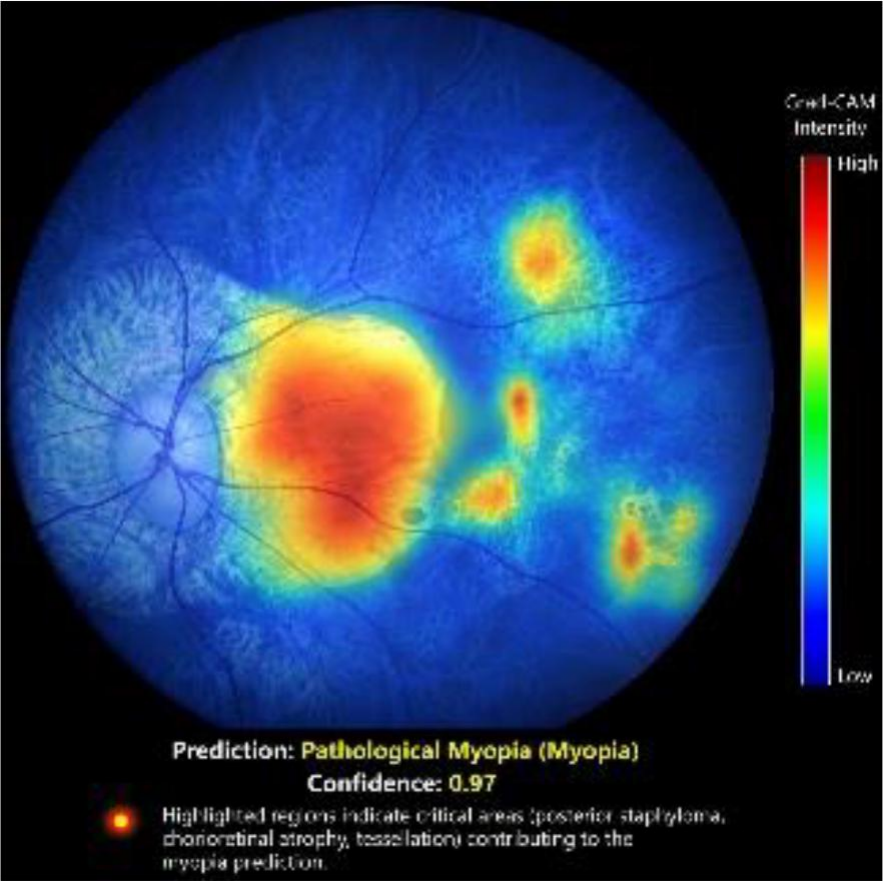	Heatmap focuses on atrophic and degenerative retinal regions, capturing structural abnormalities characteristic of pathological myopia.
Other Retinal Diseases	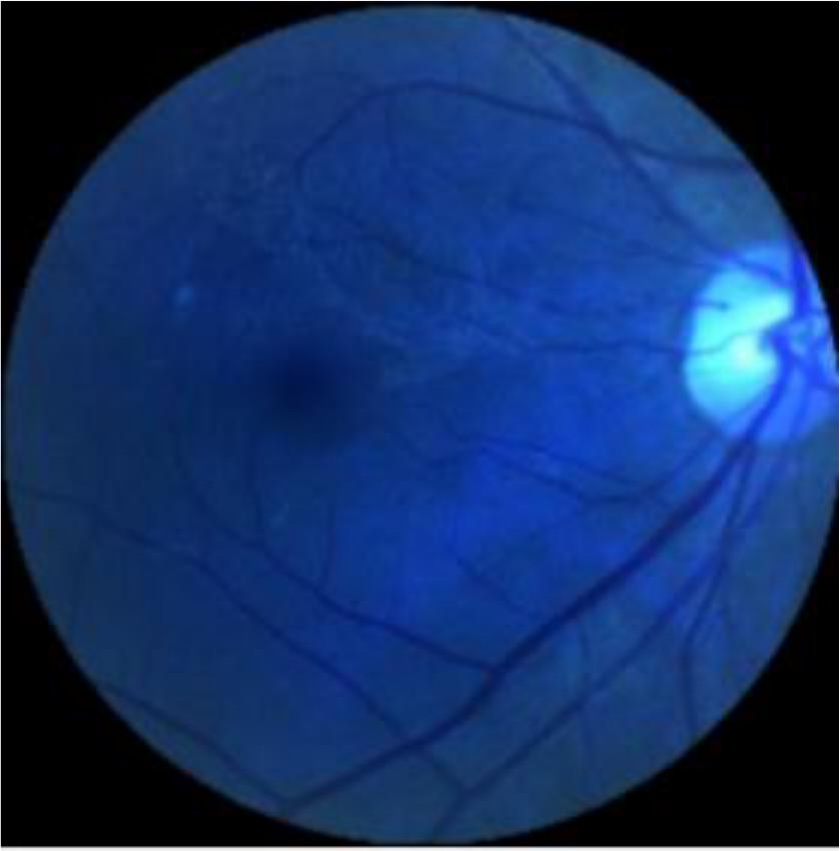	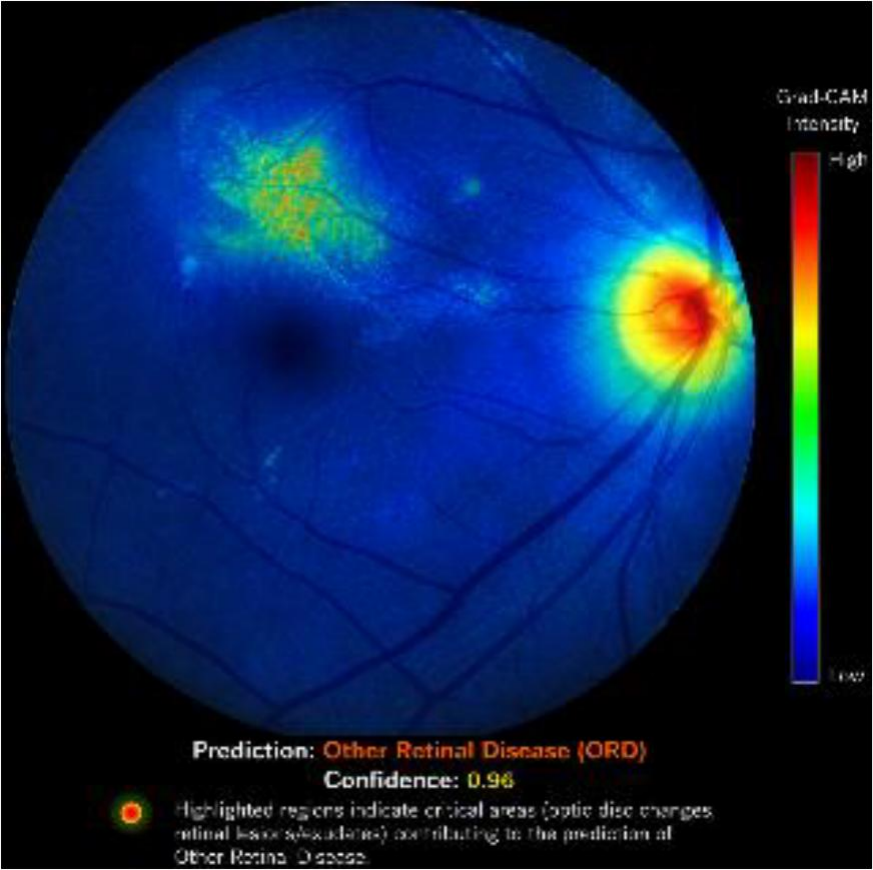	Broad activation highlights diverse lesion patterns, enabling recognition of heterogeneous retinal abnormalities beyond major classes.

## 5 Discussions

The proposed VG-RETFound framework establishes a novel paradigm for retinal disease classification by bridging retinal foundation models with anatomically informed vessel-topology learning. A major contribution of this study is the development of a novel hybrid vision impairment screening architecture that integrates RETFound-based global retinal representation learning with graph-based vessel topology modeling. Existing retinal disease classification frameworks primarily rely on image-based feature extraction and often overlook the explicit anatomical organization of the retinal vasculature. While foundation models such as RETFound provide powerful global contextual representations that can capture retinal lesions, optic disc characteristics, and macular abnormalities, they do not explicitly encode vascular connectivity and structural relationships. The proposed framework addresses this limitation by incorporating vessel-graph representations that model retinal vasculature as an anatomical network. This dual-representation strategy enables simultaneous learning of appearance-level and structural retinal information, thereby providing a more comprehensive characterization of retinal pathology. The experimental results indicate that the integration of global retinal semantics with vessel-topology information substantially improves disease discrimination, particularly for vascular-related disorders such as diabetic retinopathy, hypertensive retinopathy, and pathological myopia.

Another key innovation is the proposed cross-modal multi-head attention fusion mechanism, which facilitates dynamic interaction between RETFound image embeddings and vessel-graph representations. Unlike conventional multimodal approaches that employ simple feature concatenation or late-fusion techniques, the proposed attention-guided fusion framework learns the relative importance of vascular and appearance-based features for each disease category. By adaptively weighting interactions between retinal lesions and vascular structures, the model can identify disease-specific anatomical patterns that may not be apparent when each modality is analyzed independently. This capability closely resembles the clinical reasoning process of ophthalmologists, who simultaneously evaluate retinal lesions, vascular abnormalities, and anatomical context when making diagnostic decisions. The ablation study further demonstrates that removing the attention fusion module leads to a noticeable reduction in classification performance, confirming its critical role in learning complementary disease representations and enhancing model robustness.

The third major contribution is the introduction of an asymmetric structural fine-tuning paradigm that enables efficient adaptation of foundation-model knowledge to retinal disease classification. Existing approaches typically employ either full fine-tuning or uniform parameter-efficient tuning strategies across all network components. However, such approaches often fail to account for the distinct functional roles of individual modules within a multimodal architecture. In the proposed framework, the large-scale RETFound backbone is adapted using LoRA to preserve retinal knowledge acquired during large-scale pretraining, while the vessel-graph module, cross-attention fusion layers, and classification head are fully optimized to learn task-specific anatomical representations. This modality-aware optimization strategy reduces computational complexity, mitigates catastrophic forgetting, and allows newly introduced components to specialize in disease-relevant vascular characteristics. The superior performance observed in the ablation analysis demonstrates that the proposed fine-tuning strategy contributes significantly to both classification accuracy and model generalization, establishing an effective balance between computational efficiency and anatomical specialization.

These three innovations form the foundation of VG-RETFound and explain its performance across both internal and external evaluations. The hybrid retinal representation learning architecture provides complementary appearance and anatomical information, the cross-modal attention mechanism enables effective interaction between modalities, and the asymmetric fine-tuning strategy ensures efficient adaptation without sacrificing pretrained retinal knowledge. The combination of these contributions results in an interpretable, computationally efficient, and highly generalizable framework for automated vision impairment screening and retinal disease diagnosis. Ultimately, the study highlights the importance of incorporating domain-specific anatomical knowledge into foundation-model architectures and provides a robust foundation for future developments in deployable ophthalmic artificial intelligence systems.

Despite the promising results, several limitations deserve attention. The first limitation relates to the reliance on pretrained vessel segmentation models for graph construction. Although segmentation is performed automatically and does not require additional annotations, segmentation errors may propagate into the graph-learning pipeline and potentially affect downstream classification performance. This issue may become more pronounced in low-quality fundus images containing severe artifacts, poor illumination, motion blur, or advanced pathological changes. Another limitation is the increased computational complexity in graph construction and multimodal fusion. Though the asymmetric fine-tuning strategy reduces optimization costs compared to full foundation model training, building vessel graphs introduces an additional preprocessing step. Graph extraction can be performed offline and reused for training and inference, but large-scale screening systems may still require more computationally efficient methods for generating graphs.

Another limitation is the exclusive reliance on color fundus photography. While fundus imaging remains one of the most accessible and widely adopted modalities for retinal screening, other ophthalmic imaging techniques, including OCT, angiography, and fluorescein angiography, provide complementary structural and functional information that may further improve disease characterization. Integrating multimodal ophthalmic data could enrich disease representations and potentially improve diagnostic performance. Furthermore, the current framework focuses on static image classification and does not explicitly model disease progression or temporal changes. Longitudinal retinal imaging data may provide valuable information regarding disease evolution, treatment response, and risk stratification.

Several promising avenues for future research emerge from this study. Combining different imaging types with clinical data and electronic health records might lead to more detailed and personal diagnostic systems. Advanced graph-learning frameworks—such as hierarchical, dynamic, and hypergraph neural networks—could provide deeper insights into retinal organization and disease progression. In addition, self-supervised graph representation learning shows promise by potentially reducing reliance on preset vessel extraction methods and improving the system's ability to learn anatomical features. Additionally, techniques like model compression, knowledge distillation, and edge-aware optimization could help make lighter versions of VG-RETFound that operate on portable devices and mobile health applications.

## 6 Conclusions

This study proposes a novel framework, VG-RETFound, which combines retinal representations and retinal topology learning and is intended for automated diagnosis of vision impairment and multi-disease retinal classification from fundus images. VG-RETFound mitigates the drawbacks of state-of-the-art deep learning-based methods such as CNNs, ViTs, and foundation models for retinal disease identification, which mostly operate on appearance-driven representations and cannot account for the underlying anatomy of retinal vessels. This study uses a cross-modal multi-head attention module that enables adaptive interaction between image representations and vessel-graph embeddings, allowing complementary anatomical and semantic information to be learned jointly. Furthermore, an asymmetric fine-tuning strategy preserves the general retinal knowledge acquired during pretraining while selectively adapting the model to capture disease-specific retinal characteristics with improved parameter efficiency. The experimental results showed that the framework benefits from incorporating complementary retinal information and achieves promising performance and transferability across datasets. In particular, the use of Grad-CAM-based explanations provides clinically meaningful visualizations, which were evaluated by experienced ophthalmologists. These results highlight the importance of designing the anatomical prior into foundation model frameworks and show the efficacy of using topology information to enhance retinal disease diagnosis. While the proposed method demonstrates strong performance, it depends on the accuracy of vessel extraction and graph construction, which can be adversely affected by image artifacts, poor illumination, or pathological changes. Furthermore, the current framework works only with color fundus imaging and ignores additional multimodal ophthalmic data. Future research will focus on multimodal retinal analysis, advanced graph learning, modeling anatomical uncertainty, and developing a more efficient inference pipeline to enable real-time deployment. Overall, VG-RETFound paves the way toward next-generation ophthalmic artificial intelligence systems for mass vision impairment screening and early retinal disease detection.

## Data Availability

The original contributions presented in the study are included in the article/Supplementary Material, further inquiries can be directed to the corresponding author.
